# Revisiting the Tissue Microenvironment of Infectious Mononucleosis: Identification of EBV Infection in T Cells and Deep Characterization of Immune Profiles

**DOI:** 10.3389/fimmu.2019.00146

**Published:** 2019-02-20

**Authors:** Mário Henrique M. Barros, Gabriela Vera-Lozada, Priscilla Segges, Rocio Hassan, Gerald Niedobitek

**Affiliations:** ^1^Institute for Pathology, Unfallkrankenhaus Berlin, Berlin, Germany; ^2^Bone Marrow Transplantation Center, Instituto Nacional de Câncer, Rio de Janeiro, Brazil; ^3^Institute for Pathology, Sana Klinikum Lichtenberg, Berlin, Germany

**Keywords:** Epstein-Barr virus, infectious mononucleosis, tissue microenvironment, PD-L1, EBV+ T cells, macrophage polarization

## Abstract

To aid understanding of primary EBV infection, we have performed an in depth analysis of EBV-infected cells and of local immune cells in tonsils from infectious mononucleosis (IM) patients. We show that EBV is present in approximately 50% of B-cells showing heterogeneous patterns of latent viral gene expression probably reflecting different stages of infection. While the vast majority of EBV+ cells are B-cells, around 9% express T-cell antigens, with a predominance of CD8+ over CD4+ cells. PD-L1 was expressed by a median of 14% of EBV+ cells. The numbers of EBER+PD-L1+ cells were directly correlated with the numbers of EBER+CD3+ and EBER+CD8+ cells suggesting a possible role for PD-L1 in EBV infection of T-cells. The microenvironment of IM tonsils was characterized by a predominance of M1-polarized macrophages over M2-polarized cells. However, at the T-cell level, a heterogeneous picture emerged with numerous Th1/cytotoxic cells accompanied and sometimes outnumbered by Th2/regulatory T-cells. Further, we observed a direct correlation between the numbers of Th2-like cells and EBV– B-cells. Also, a prevalence of cytotoxic T-cells over Th2-like cells was associated with an increased viral load. These observations point to contribution of B- and Th2-like cells to the control of primary EBV infection. 35% of CD8+ cells were differentiated CD8+TBET+ cells, frequently detected in post-capillary venules. An inverse correlation was observed between the numbers of CD8+TBET+ cells and viral load suggesting a pivotal role for these cells in the control of primary EBV infection. Our results provide the basis for a better understanding of immune reactions in EBV-associated tumors.

## Introduction

Epstein-Barr virus (EBV) infects more than 90% of adults worldwide and persists in memory B lymphocytes as a life-long asymptomatic infection (1). Primary EBV infection in its natural setting (that is early in life) is mostly asymptomatic while in adolescents and young adults, 20–50% of primary infections lead to the clinical syndrome of infectious mononucleosis (IM), which is an EBV-driven proliferation of B-lymphocytes that is controlled by humoral and cellular immune responses (1, 2). In IM, characteristically, there is a florid T cell response mainly consisting of activated CD8+ cytotoxic T cells specific for lytic, and to a lesser extent, latent viral antigens expressed in EBV-infected B-cells (1, 3). Knowledge about this response, with its particular antigenic hierarchy and dynamics has been accumulating during the last decades (4, 5).

However, in contrast to the vast amount of knowledge derived from studies focusing on EBV-infected cells, immune cells and antibody response in peripheral blood (1, 3, 6–11), only few studies have focused on the characteristics of EBV infection and the tissue microenvironment response at the main anatomical site of primary virus infection (6, 12–15) and detailed information as to what happens in the tonsils of these patients is still wanting. For example, while the magnitude of EBV infected B cells in the peripheral blood is known (16, 17) the proportion of B cells infected by EBV in tonsils at the peak of the primary EBV infection is unknown. Moreover, quantity and composition of the local immune cell response, the relation between the magnitude of EBV-infection and the local immune response signature, and the extent to which the immune response observed in the peripheral blood is reflected in the tissue microenvironment remain unexplored.

Hierarchical immune response against EBV is driven by the specific patterns of EBV latent and lytic cycle gene expression during primary infection and establishment of persistence (5). The restricted latency I pattern is characterized by the expression of EBV-encoded nuclear RNAs (EBERs) and the virus-encoded nuclear antigen (EBNA) 1 only. In the latency IIa, there is the expression of EBERs, EBNA1 and latent membrane protein (LMP) 1, while in the latency IIb pattern EBERs and EBNA1 are expressed together with EBNA2 but not LMP1. In the latency III pattern, EBERs and all the viral latent proteins are expressed: EBNA1, EBNA2, EBNA3A, EBNA3B, EBNA3C, EBNALP, LMP1, LMP2A, and LMP2B (18, 19). These proteins display hierarchical immunodominance for the CD8+ T cell response and the strongest responses are induced by EBNA3A, EBNA3B, and EBNA3C (5, 20). A similar hierarchical response pattern is observed for the lytic cycle antigens, to which the strongest responses are observed against the immediate early antigens BZLF1 and BRLF1 (5).

EBV infection is usually kept in check by the immune system; therefore, persistence does not result in clinical symptoms in most individuals. However, under certain circumstances, EBV is associated with the development of EBV-associated lymphomas (1), the majority B-cell derived (21). In addition, some T-cell lymphomas are associated with EBV, such as various T/NK cell lymphoproliferative disorders of childhood and nasal-type extranodal T/NK cell lymphomas (21). It is uncertain when exactly EBV infection occurs in the pathogenesis of these T/NK cell disorders. While it is generally accepted that in the immunocompetent host, EBV infection in the pathogenesis of T-cell lymphomas takes place prior to the clonal expansion of neoplastic cells (22), it is uncertain if it occurs during primary EBV infection or later during viral persistence. At present, information on the infection of T or NK cells by EBV during primary infection is scarce (23).

Since symptomatic primary infection itself has been associated with the development of neoplastic (24) and autoimmune diseases (25), knowledge of the events that occur during IM would be important for understanding the factors influencing the risk of EBV-associated diseases development. In this study we have therefore carried out an *in situ* analysis of a series of IM tonsils to characterize EBV infection, tissue microenvironment composition and immune response signature.

## Methods

### Tissues

Formalin-fixed paraffin-embedded (FFPE) tissue blocks from 16 tonsils with a diagnosis of IM were included. All patients were submitted to tonsillectomy for severe obstructive tonsillitis. Age ranged from 7 to 31 years (median 20 years). For analysis, patients were categorized in two age groups (≤19 years and ≥20 years). Fourteen cases (87.5%) were male and 2 cases (12.5%) female.

All cases were selected from the archives of the Institute of Pathology, Unfallkrankenhaus Berlin. All materials were submitted for diagnostic purposes and were anonymised. No tissue samples have been collected solely for the purpose of this study. The FFPE tissue blocks were used in accordance with national ethical principles and Declaration of Helsinki, dispensing a compulsory statement from an ethics committee, according to local and national guidelines. All histological diagnoses were reviewed before inclusion in this study.

A Tissue arrayer device (Beecher Instrument, Estonia/USA) was used to assemble the tissue microarray (TMA) blocks. From each case, four 2-mm-diameter cores selected from four different areas rich in EBER+ cells were included. To ascertain that the cores contained representative numbers of EBV-infected cell, all TMAs were subjected to EBER-specific *in situ* hybridization again (EBER-ISH, see below). All cases showed cores with high numbers of EBER+ cells/mm^2^ (from 105 to 1,006 EBER+ cells/mm^2^, median: 390 cells/mm^2^).

### EBV Detection

Latent EBV infection was determined in all cases by *in situ* hybridization (ISH) for EBERs (EBER-ISH) as described previously (26), employing diaminobenzidine (DAB) chromogen (Zytomed Systems, Berlin, Germany) as chromogen. The latent proteins were evaluated by immunohistochemistry (IHC) as described previously, using the antibodies against EBNA1 (clone 1H4, kind gift from Dr. Kremmer, Munich, Germany), EBNA2 (clone PE2, kind gift from Dr. M. Rowe, Birmingham, UK), LMP1 (clones CS1-4, Zytomed Systems) and BZLF1 (clone BZ1, Santa Cruz, Dallas, USA) (27).

### Double EBER-ISH and Immunohistochemistry

To evaluate the number of B cells infected by EBV, a double EBER-ISH and IHC assay was used to discriminate EBV-infected B cells (EBER+CD20+) from EBV-negative B cells (EBER– CD20+). Following completion of the EBER-ISH assay as described above, antigen retrieval was immediately performed by heat treatment in a pressure-cooker for 1 min, using citrate buffer pH 7.6. A blocking step was conducted, using Blocking Solution included in the AP Polymer System (Zytomed Systems), according to the manufacturer's instructions. Anti-CD20 was used as primary antibody and was incubated overnight in a wet chamber at 4°C. Following the manufacturer's instructions, immunodetection was performed with AP Polymer System (Zytomed Systems) in a wet chamber at room temperature, employing Vector Blue Alkaline Phosphatase Substrate Kit III (Vector Laboratories) under microscopic control until optimal blue staining is reached (circa 10 min). Subsequently the slides were washed in distilled water for 5 min and immediately mounted using Glycergel Mounting Medium (DakoCytomation, Santa Clara, California, USA). Sections were not counterstained. For evaluation of numbers of EBV-negative CD20+ B cells/mm^2^ in the IM microenvironment, only EBER– CD20+ cells were counted. Similarly, a double EBER-ISH and IHC assay was also used to evaluate the expression of non B-cell markers on EBER+ cells. For this purpose, CD3, CD4, CD8, CD56, CD68, and CD83 antibodies were used in the IHC step. For a complete list of antibodies see Table S1.

### Immune Cell Populations in the Microenvironment

A single labeling immunohistochemical methodology previously described (28) was used to study cell subpopulations, using anti- CD3, CD4, FOXP3, CD8, TIA1, Granzyme B and CD56 primary antibodies (28). Buffers used for antigen retrieval and primary antibodies are listed in the (Table S1).

A double labeling IHC method was employed to study the expression of TBET and CMAF by CD4+ cells, the expression of TBET by CD8+ cells, markers of macrophage polarization as described previously (29, 30) and to establish the prevalent EBV latency patterns. Briefly, the antibodies specific for nuclear antigens (TBET, CMAF, pSTAT1, or EBNA2) were used as first primary antibodies and the detection of bound antibodies was performed using ZytoChem Plus HRP polymer kit (Zytomed Systems, Berlin, Germany), employing DAB as chromogen. The antibodies directed against membranous antigens (CD4, CD8, CD68, CD163, or LMP1) were applied subsequently, followed by detection with AP Polymer System (Zytomed Systems), employing Blue Alkaline Phosphatase substrate kit (Vector Laboratories, CA, USA). Sections were not counterstained.

To evaluate if differentiated CD8+TBET+ cells can be observed in different compartments of the tissue microenvironment of IM, a triple labeling IHC assay to identify CD8+TBET+ cells, blood vessels, lymphatic vessels and epithelium was performed. Following double labeling IHC to identify CD8+TBET+ cells as described above, the slides were washed and incubated with antibodies specific for Factor VIII (clone EP3372, Zytomed Systems) to identify blood vessel endothelium, CK5/6 (clone D5/16B4, Zytomed Systems) to identify epithelial cells that cover the tonsils or Podoplanin (clone D2-40, BioLegend, San Diego, USA) to identify lymphatic endothelium. For detection of immobilized antibodies, AP Polymer System (Zytomed Systems) was used, employing Permanent AP Red as chromogen (Zytomed Systems). Sections were not counterstained.

### PD-L1 Expression

A double EBER-ISH and IHC assay was used to evaluate the expression of Programmed Death 1 ligand (PD-L1) (clone QR1, Quartett, Berlin, Germany) in EBER+ cells. Only EBER+ cells that expressed strong and unequivocal PD-L1 membranous staining were counted. For comparison and to assess the distribution pattern of PD-L1-expressing cells in tonsils, we performed a single labeling IHC using whole tissue sections of 3 tonsils with follicular hyperplasia and of 3 tonsils with IM.

### Thresholds

Computer assisted microscopical analysis was performed as described previously (28, 31). For comparison of two cell populations, a predominance of one cell population over the other was considered when cell numbers were at least 1.5x higher, as described previously (29, 30) (Figure S1).

### EBV Genotyping and Viral Load

DNA was extracted from FFPE whole sections of IM tonsils included in this study with ReliaPrep FFPE gDNA Miniprep System (Promega). Genotyping was carried out by specific PCR assays to distinguish EBV-1 and−2 EBNA2 and EBNA3C regions, as described (32). For viral load quantification, a specific qPCR assay designed to amplify a region of EBV BNT/p143 gene and β2-microglobulin was used, as described (33). Reactions were set up with final volumes of 20 μl, containing 900 nM of primers and 200 nM of Taqman FAM-MGB probe (Applied Biosystems by Life Technologies). qPCR reactions were performed in duplicate in a Viia7 Real-time PCR system (Applied Biosystems by Life Technologies) with the following cycling parameters: 2 min at 50°C, 10 min at 95°C, and 50 cycles of 15 s at 95°C and 60 s at 60°C. Quantification was performed by reverse calibration against a standard curve, constructed with serial dilutions of a commercially quantified control (Amplirun Epstein Barr virus DNA control, Vircell), ranging from 5 to 5 × 10^6^ genome equivalents (g/Eq); and an in-house recombinant plasmid (pCR 2.1, Invitrogen) containing a fragment of the β2-microglobulin (*B2MG*) gene, ranging from 5 to 5 × 10^5^ copies /reaction. Both logarithmical dilution series were prepared in TE-dissolved solution of 100 ng/mL of yeast tRNA (Ambion by Life Technologies). Normalized EBV quantities were calculated as: EBV copy number/diploid cell number × 100,000 cells = (EBV copy number)/(B2MG number/2) × 100,000 cells.

### Statistical Analysis

To verify association between variables, Pearson's chi-square, Fisher's exact test, Mann-Whitney test and Spearman's rank correlation were used as described previously (28, 29, 31, 34, 35). Data were analyzed using SPSS 13.0. All possible correlations were tested for all cell populations analyzed in this study. Only the ones showing statistical significance are described in the results. All the evaluated correlations are described in the Table S2.

## Results

### EBV Infection

**Figure 1:**
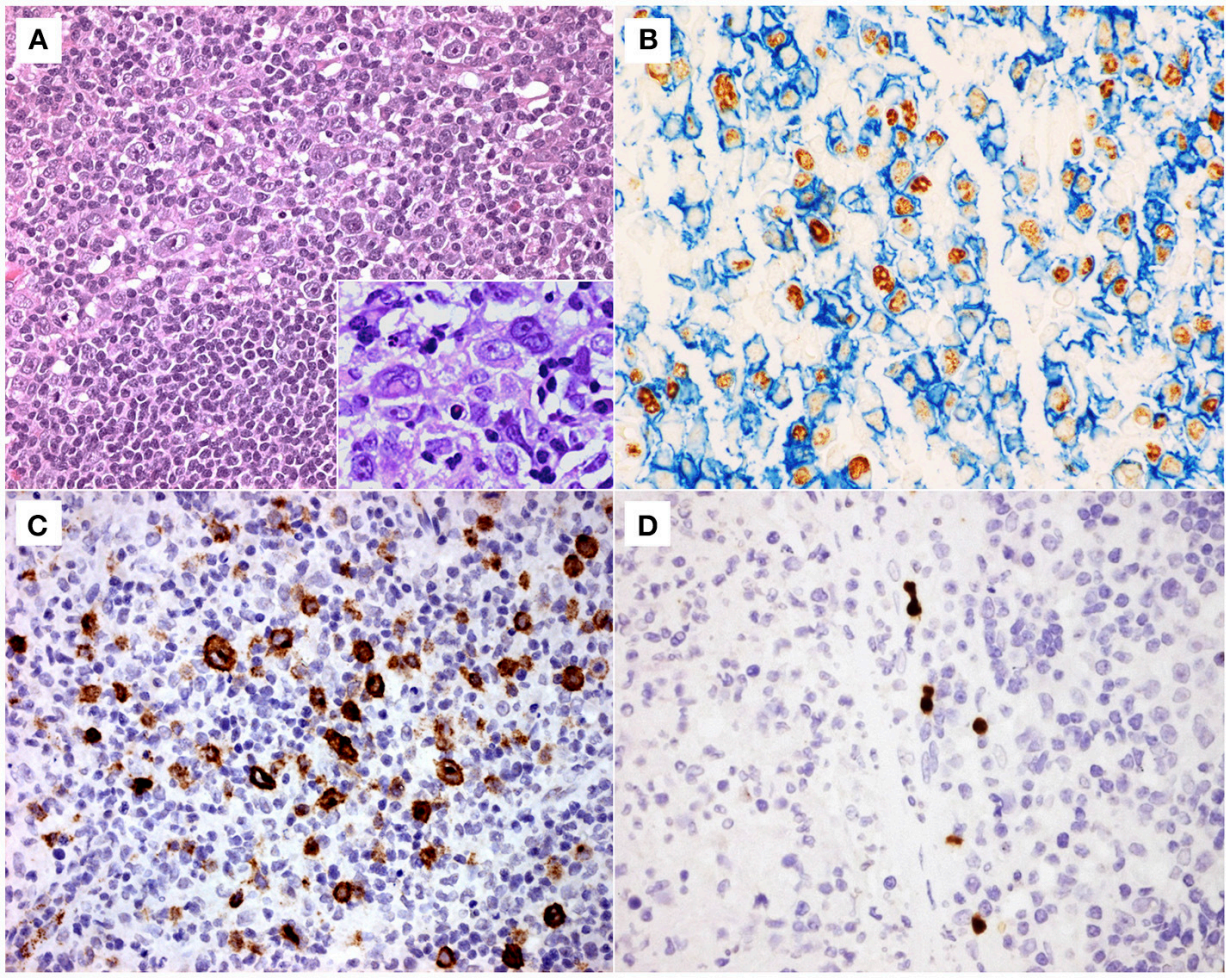
**(A)** Histological section stained with haematoxylin and eosin of a representative case of infectious mononucleosis (IM), exhibiting a polymorphous lymphoid cell hyperplasia with small lymphocytes, immunoblasts and Reed-Sternberg (RS)-like cells (original magnification 200x). In detail, immunoblasts and RS-like cells (original magnification 400x). **(B)** Double labeling EBER-specific *in situ* hybridization and CD20 immunohistochemistry showing numerous B cells (membranous blue staining) infected by Epstein-Barr virus (nuclear brown staining) (original magnification 400x). **(C)** A representative case of IM showing many LMP1+ cells (membranous brown staining) (original magnification 400x) and **(D)** a few BZLF1+ cells (nuclear brown staining) (original magnification 400x).

EBER+ cells with morphology of large, blast-like lymphoid cells including Hodgkin and Reed-Sternberg (HRS)-like cells were detected in all the cases (Figure 1). The majority of these cells were predominantly found in the interfollicular regions, although small numbers of EBER+ cells were also observed in the germinal centers and within the tonsillar crypt epithelium.

The numbers of EBER+ cells/mm^2^ were variable (from 105 to 1006 cells/mm^2^, median 390 cells/mm^2^). The numbers of EBER+ cells expressing CD20 (EBER+CD20+) were lower (20–668 cells/mm^2^, median 219 cells/mm^2^), suggesting that not all EBV-infected cells express CD20 (Table 1). The viral load was also variable (from 642 copies/10^6^ cells to 74,014 copies/10^6^ cells, median: 19,172 copies/10^6^ cells). All cases harbored type 1 EBV (EBV-1).

**Table 1:** Characterization of EBER+ cells and latent viral proteins in the tissue microenvironment of 16 cases with infectious mononucleosis.

**Cells**	**Range (cells/mm^**2**^)**	**Median (cells/mm^**2**^)**
EBER+	105–1,006	390
EBER+CD20+	20–668	219
EBER+CD20–	5–545	105
EBER+CD3+	0–26	8
EBER+CD4+	3–29	8
EBER+CD8+	0–23	8
EBER+CD83+	3–108	41
EBER+PDL1+	11–117	41
EBNA1+	143–645	358
EBNA2+	57–563	210
LMP1+	22–184	52
EBNA2+LMP1+	20–171	43
EBNA2+LMP1–	14–420	150
EBNA2–LMP1+	3–171	72
BZLF1+	0–38	5

*Formalin-fixed paraffin-embedded tissue blocks of tonsils were used in all experiments (see methods for detailed description)*.

Analyzing the latent viral proteins, we first noticed that overall, the numbers of EBNA1+ cells were lower than that of EBER+ cells, suggesting that EBNA1 immunohistochemistry is less sensitive than EBER ISH for the detection of latently infected cells. Further, we observed a prevalence of EBNA1+ cells (143–645 cells/mm^2^; median: 357 cells/mm^2^) over EBNA2+ cells (57–563 cells/mm^2^; median: 210 cells/mm^2^) and LMP1+ cells (22–184 cells/mm^2^; median: 52 cells/mm^2^). Only a small fraction of EBV+ cells was in lytic cycle as disclosed by BZLF1 expression (0–38 cells/mm^2^; median 5 cells/mm^2^) (Table 1).

A double IHC approach, based on the simultaneous detection of EBNA2 and LMP1 was used to define latency patterns (other than latency I), namely latency III (EBNA2+ LMP1+), latency IIa (EBNA2- LMP1+) and latency IIb (EBNA2+ LMP1-), as described (18). The majority of EBV+ cells exhibited latency IIb pattern (EBNA2+LMP1-, 14–420 cells/mm^2^, median 150 cells/mm^2^), followed by latency IIa pattern (EBNA2- LMP1+, 3–171 cells/mm^2^, median 72 cells/mm^2^) and latency III pattern (EBNA2+LMP1+, 20–171 cells/mm^2^, median 43 cells/mm^2^) (Table 1). All these different latency patterns were observed co-existing randomly in the interfollicular regions and germinal centers without a specific distribution pattern, as described previously (14). Neither in our current nor in previous studies there was any evidence of an expansion of EBV-infected cells in germinal center reactions (14). There was no cluster formation of EBV+ cells expressing the same latency pattern (Figure 1). Many LMP1 expressing cells showed a Hodgkin- and Reed-Sternberg-like morphology (Figure 1).

The total numbers of EBER+ cells/mm^2^ were directly correlated with the numbers of EBER+CD20+ cells/mm^2^ (rho = 0.8, *P* < 0.0005; Spearman's correlation), EBNA2+ cells/mm^2^ (rho = 0.6, *P* = 0.007; Spearman's correlation) and the viral load (rho = 0.6, *P* = 0.009; Spearman's correlation). However, unexpectedly, the numbers of EBER+ cell/mm^2^ were not correlated with numbers of EBNA1+ cells/mm^2^ (rho = 0.2, *P* = 0.4; Spearman's correlation) or LMP1+ cells/mm^2^ (rho = −0.1, *P* = 0.5; Spearman's correlation).

The numbers of EBNA1+ cells were directly correlated with the numbers of EBNA2+ cells/mm^2^ (rho = 0.5, *P* = 0.03; Spearman's correlation) and with the numbers of latency IIb EBNA2+LMP1- cells/mm^2^ (rho = 0.57, *P* = 0.03; Spearman's correlation). However, an inverse correlation was observed between the numbers of EBNA1+ cells/mm^2^ and latency IIa EBNA2-LMP1+ cells/mm^2^ (rho = −0.66, *P* = 0.007; Spearman's correlation). There was no correlation between the numbers of EBER+ cells and the numbers of cells displaying latencies IIa, IIb, or III, respectively.

The numbers of EBV-infected cells in lytic cycle expressing BZLF1 were not correlated with any parameter indicating the magnitude of EBV-infection and with any kind of latency pattern. However, a trend to higher numbers of EBER+ cells/mm^2^ and higher viral load was observed in patients with until 19 years (*P* = 0.059 and *P* = 0.05, respectively; Mann-Whitney test). No other correlations were observed considering the age groups (Figure S2).

### Are All EBER+ Cells B Cells?

**Figure 2:**
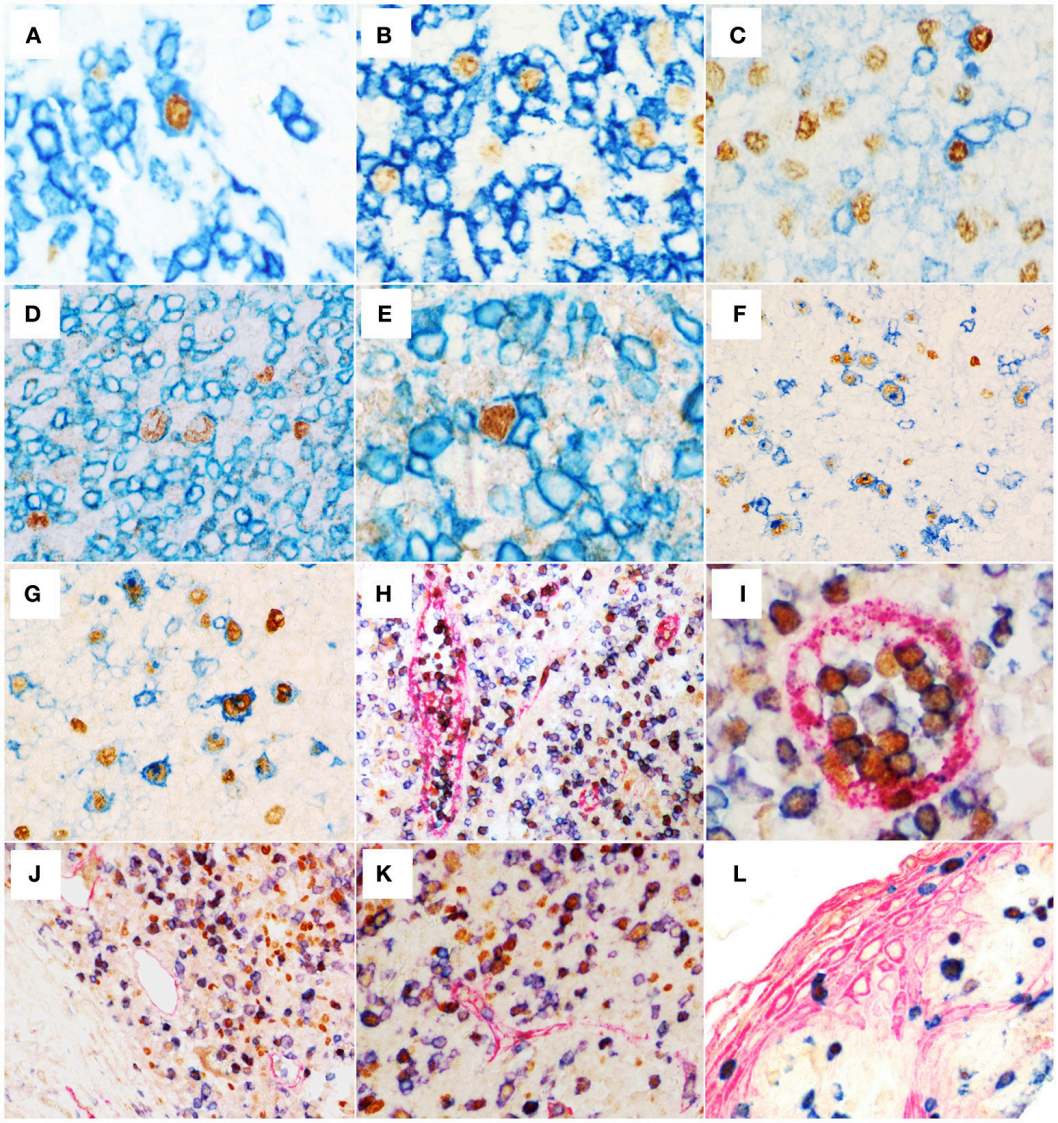
Double labeling EBER-specific *in situ* hybridization (nuclear brown staining) and immunohistochemistry (IHC) for the detection of CD3 **(A)**, CD8 **(B)**, and CD4 **(C)** (membranous blue staining) (original magnification 600x) reveals co-localization of signals indicating EBV-infection of occasional T-cells. Double labeling IHC showing expression of viral proteins EBNA1 **(D)** and EBNA2 **(E)** (nuclear brown staining) in CD3+ cells (membranous blue staining) (original magnification 600x). **(F)** Double labeling EBER-specific *in situ* hybridization (nuclear brown staining) and CD83-specific IHC (membranous blue staining) with numerous EBER+CD83+ cells (original magnification 600x). **(G)** Double labeling IHC for the detection of PAX5 (nuclear brown staining) and CD83 (membranous blue staining) showing that the majority of CD83+ cells were also PAX5+ B cells (original magnification 600x). **(H–K)** Triple IHC for the detection of CD8 (membranous blue staining), TBET (nuclear brown staining), Factor VIII (membranous red staining in **H,I**) or Podoplanin (membranous red staining in **J,K**). Note that large numbers of differentiated CD8+TBET+ cells are found in the lumina of post-capillary venules (H and I, in red, original magnification 400x and 600x, respectively) but not in the in the lymphatic vessels (**J,K**, in red, original magnification 400x). **(L)** Triple IHC for the detection of CK5/6 (membranous red staining), CD8 (membranous blue staining), and TBET (nuclear brown staining) showing that differentiated intraepithelial CD8+TBET+ cells can be observed in infectious mononucleosis tonsils (original magnification 400x).

To evaluate the presence of EBV-infected cells other than B cells, we performed double EBER-ISH/IHC assays. First, the numbers of EBER+ cells negative for CD20 were assessed and a median of 105 EBER+CD20- cells/mm^2^ was observed (5–545 cells/mm^2^). Subsequently, EBER ISH was combined with the detection of T cell-specific antigens. All cases showed a variable proportion of T cells infected by EBV with median of 8 EBER+CD3+ cells/mm^2^ (0–26 cells/mm^2^), median of 8 EBER+CD4+ cells/mm^2^ (3–29 cells/mm^2^), and median of 8 EBER+CD8+ cells/mm^2^ (0–23 cells/mm^2^) (Figure 2, Table 1). Morphologically, these cells were usually small to medium-sized lymphocytes, although immunoblast-like cells were also observed.

Considering the percentage of EBER+ cells expressing T cell antigens, we observed a median of 9.1% of EBER+CD3+ cells (0–30%), 3.6% of EBER+CD4+ cells (2.53–23.77%) and 6% of EBER+CD8+ cells (0–31%). The absolute numbers of EBER+CD3+, EBER+CD4+, and EBER+CD8+ T cells did not correlate with the numbers of EBER+ cells, EBNA1+ cells, EBER+CD20+ cells, EBNA2+ cells, LMP1+ cells and the viral load.

The expression of EBNA1, EBNA2 and BZLF1 by EBV+CD3+ cells were evaluated by double immunohistochemistry assays in whole tissue sections of 3 cases that exhibited the highest numbers of EBV-infected T cells. In all cases, EBV+CD3+ cells expressing EBNA1 and EBNA2 were observed, indicating latency II or III patterns (Figure 2). For technical reasons, it was not possible to evaluate the expression of LMP1 in these cells and therefore no further discrimination of latency patterns was possible using our approach. BZLF1 was not expressed by the CD3+ cells in any of these cases.

In an attempt to evaluate the infection of dendritic cells by EBV, we also analyzed the expression of the dendritic cell marker CD83 by the EBER+ cells. A median of 41 EBER+CD83+ cells/mm^2^ (3–108 cells/mm^2^) and a median of 76 EBER– CD83+ cells/mm^2^ (14–272 cells/mm^2^) were observed (Figure 2). On account of the high numbers of EBER+CD83+ cells observed and a report suggesting that CD83 can be aberrantly expressed by EBER+ B cells *in vitro* (36), we decided to perform a double IHC using CD83 and PAX5. As shown in the Figure 2, many CD83+ cells were also PAX5+. A direct correlation was observed between the numbers of EBER+CD83+ and PAX5+CD83+ cells/mm^2^ (rho = 0.6, *P* = 0.008; Spearman's correlation), while the numbers of EBER– CD83+ and PAX5+CD83+ cells/mm^2^ were not correlated (rho = 0.4, *P* = 0.1; Spearman's correlation). These results reinforce the notion that CD83 is aberrantly expressed in EBV-infected B-cells in IM and indicate that the identification of dendritic cells in IM should not rely on CD83 antibody alone. In support of this, we did not observe any EBER+S100+ dendritic cells in any of our cases (not shown).

Interestingly, the numbers of EBER+CD83+ cells were directly correlated only with the numbers of LMP1+ cells/mm^2^ (rho = 0.57, *P* = 0.02; Spearman's correlation). No correlation was observed between the numbers of EBER– CD83+ and LMP1+ cells/mm^2^ (rho = −0.1, *P* = 0.6, Spearman's correlation). No unequivocal expression of CD56 or CD68 in EBER+ cells was observed (not shown).

### Tissue Microenvironment in IM

#### Lymphocyte Populations

In the IM tissue microenvironment, CD3+ T cells were more prevalent than EBV– B cells (median 812 cells/mm^2^ for T cells and 258 cells/mm^2^ for EBV– B cells, respectively). From the total CD20+ B cells in the IM tissue microenvironment, a median of 49.42% were EBER+ (5.31–76.52%). A detailed description of results is provided in Table 2.

**Table 2:** Evaluation of immune cell populations in the tissue microenvironment of infectious mononucleosis.

**Cells**	**Range (cells/mm^**2**^)**	**Median (cells/mm^**2**^)**
CD3+	413–1,410	812
CD4+	163–1,325	625
CD4+CMAF+^a^	167–1,671	543
FOXP3+^b^	35–363	132
CD4+TBET+^c^	20–91	58
CD8+	442–903	627
CD8+TBET-^d^	155–583	372
CD8+TBET+^e^	76–407	209
TIA1+^f^	120–569	335
Granzyme B+^f^	246–572	378
EBER–CD20+^g^	93–803	258
CD68+pSTAT1+^h^	35–190	115
CD68+CMAF+^i^	0–79	23
CD163+pSTAT1+^h^	29–272	99
CD163+CMAF+^i^	14–137	49
CD56+	1–20	8
EBER–CD83+	14–272	76
PAX5+CD83+^j^	3–272	93

a *Th2-like cells*.

b *Regulatory T cells*.

c *Th1-like cells*.

d *Not terminal differentiated CD8+ T cells*.

e *Terminal differentiated CD8+ T cells*.

f *Cytotoxic cells*.

g *EBV-negative B cells*.

h M1-like macrophage

i *M2-like macrophage*.

j *Assumed as EBV+ cells expressing CD83*.

Among T cells, similar numbers of CD4+ and CD8+ cells were observed (median 625 cells/mm^2^ and 627 cells/mm^2^, respectively). Among the CD4+ cells, Th2-like (CD4+CMAF+) cells were by far the most frequent subset followed by regulatory T (FOXP3+) cells and Th1-like (CD4+TBET+) cells (median 543 cells/mm^2^, 132 cells/mm^2^, and 58 cells/mm^2^, respectively). See Table 2 for detailed range description. Relatively large numbers of cells expressing the cytotoxic markers TIA1 and Granzyme B were observed (median 335 and 378 cells/mm^2^, respectively) (Table 2).

Analyzing correlations between immune cell subpopulations, a direct correlation between the numbers of Th1-like and CD8+ cells (rho = 0.54, *P* = 0.029; Spearman's correlation) was observed, as expected. Furthermore, there were inverse correlations of the numbers of regulatory T cells and cytotoxic TIA1+ cells (rho = −0.57, *P* = 0.02; Spearman's correlation), as well as of numbers of Th2-like and cytotoxic Granzyme B+ cells (rho = −0.6, *P* = 0.011; Spearman's correlation). Interestingly, the numbers of EBER– CD20+ were directly correlated with the numbers of Th2-like cells (rho = 0.67, *P* = 0.006; Spearman's correlation). A trend to higher numbers of CD3+ cells/mm^2^ was observed in patients until 19 years, compared with older patients (*P* = 0.064, Mann-Whitney test, Figure S2). No other correlations were observed.

The evaluation of ratios of immune cell populations, an exploratory statistical strategy that can reflect the microenvironment signature (Figure S1), disclosed a complex scenario. In line with what was expected, 100% of cases showed more CD8+ than regulatory T (FOXP3+) cells, 93.8% of cases (15/16) exhibited more cytotoxic Granzyme B+ than regulatory T cells, and 81.3% of cases (13/16) showed more cytotoxic TIA1+ than regulatory T cells (Table 3) suggesting a predominant cytotoxic signature. However, arguing against this notion, 10 of 16 cases (62.5%) exhibited less cytotoxic TIA1+ cells than Th2-like (CD4+CMAF+) cells and 11 of 16 cases (68.8%) displayed less cytotoxic Granzyme B+ cells than Th2-like cells. Regarding CD8+ and Th2-like (CD4+CMAF+) cells, similar numbers were observed in 8 of 16 cases (50%), more CD8+ than Th2-like cells were noticed in 6 of 16 cases (37.5%) and less CD8+ than Th2-like cells were present in 2 of 16 cases (12.5%) (Table 3). Evaluating the ratios of subtypes of CD4+ cells a regulatory/Th2 microenvironment was highlighted: all cases showed more Th2-like than Th1-like cells and 15 of 16 cases (93.8%) displayed more regulatory T than Th1-like cells (Table 3).

**Table 3:** Ratios of the evaluated immune cells from the tissue microenvironment of infectious mononucleosis.

**Ratio^a^**	**Number of cases (%)**
**REGULATORY T (FOXP3+) CELLS: TH1 (CD4+TBET+) CELLS**	
FOXP3+ < TBET+	1 (6.3)
FOXP3+ > TBET+	15 (93.8)
FOXP3 ≈ TBET	0
**REGULATORY T (FOXP3+) CELLS: CD8+ CELLS**	
FOXP3+ < CD8+	16 (100)
FOXP3+ > CD8+	0
FOXP3+ ≈ CD8+	0
**REGULATORY T (FOXP3+) CELLS: DIFFERENTIATED CD8+ (TBET+)**	
**CELLS**	
FOXP3+ < CD8+TBET+	13 (81.3)
FOXP3+ > CD8+TBET+	0
FOXP3+ ≈ CD8+TBET+	3 (18.8)
**REGULATORY T (FOXP3+) CELLS: NON-DIFFERENTIATED CD8+ (TBET–)**	
**CELLS**	
FOXP3+ < CD8+TBET–	14 (87.5)
FOXP3+ > CD8+TBET–	0
FOXP3+ ≈ CD8+TBET–	2 (12.5)
**REGULATORY T (FOXP3+) CELLS: CYTOTOXIC (TIA1+) CELLS**	
FOXP3+ < TIA1+	13 (81.3)
FOXP3+ > TIA1+	1 (6.3)
FOXP3+ ≈ TIA1+	2 (12.5)
**REGULATORY T (FOXP3+) CELLS: GRANZYME B+ CYTOTOXIC CELLS**	
FOXP3+ < Granzyme B+	15 (93.8)
FOXP3+ > Granzyme B+	0
FOXP3+ ≈ Granzyme B+	1 (6.3)
**TH2 (CD4+CMAF+) CELLS: TH1 (CD4+TBET+) CELLS**	
CD4+CMAF+ < CD4+TBET+	0
CD4+CMAF+ > CD4+TBET+	16 (100)
CD4+CMAF+ ≈ CD4+TBET+	0
**TH2 (CD4+CMAF+) CELLS: CYTOTOXIC (TIA1+) CELLS**	
CD4+CMAF+ < TIA1+	3 (18.8)
CD4+CMAF+ > TIA1+	10 (62.4)
CD4+CMAF+ ≈ TIA1+	3 (18.8)
**TH2 (CD4+CMAF+) CELLS: CYTOTOXIC (GRANZYME B+) CELLS**	
CD4+CMAF+ < Granzyme B+	3 (87.5)
CD4+CMAF+ > Granzyme B+	11 (68.8)
CD4+CMAF+ ≈ Granzyme B+	2 (12.5)
**TH2 (CD4+CMAF+) CELLS: CD8+ CELLS**	
CD4+CMF+ < CD8+	6 (37.5)
CD4+CMAF+ > CD8+	2 (12.5)
CD4+CMAF+ ≈ CD8+	8 (50)
**TH2 (CD4+CMAF+) CELLS: DIFFERENTIATED CD8+ (TBET+) CELLS**	
CD4+CMF+ < CD8+TBET+	0
CD4+CMAF+ > CD8+TBET+	16 (100)
CD4+CMAF+ ≈ CD8+TBET+	0
**TH2 (CD4+CMAF+) CELLS: NON-DIFFERENTIATED CD8+ (TBET–) CELLS**	
CD4+CMF+ < CD8+TBET–	2 (12.5)
CD4+CMAF+ > CD8+TBET–	12 (75)
CD4+CMAF+ ≈ CD8+TBET–	2 (12.5)
**CD4+ CELLS: CD8+ CELLS**	
CD4+ < CD8+	5 (31.3)
CD4+ > CD8+	3 (18.8)
CD4+ ≈ CD8+	8 (50)
**CD3+ CELLS: EBER– CD20+ CELLS**^b^	
CD3 > EBER– CD20+	13 (86.7)
CD3 < EBER– CD20+	0
CD3 ≈ EBER– CD20+	2 (13.3)
**CD4+ CELLS: EBER– CD20+ CELLS**^b^	
CD4 < EBER– CD20+	1 (6.7)
CD4 > EBER– CD20+	10 (66.7)
CD4 ≈ EBER– CD20+	4 (26.7)
**CD3+ CELLS: EBER– CD20+ CELLS**^b^	
CD3+ < EBER– CD20+	0
CD3+ > EBER– CD20+	13 (86.7)
CD3+ ≈ EBER– CD20+	2 (13.3)
**TH1 (CD4+TBET+) CELLS: EBER– CD20+ CELLS**^b^	
CD4+TBET+ < EBER– CD20+	15 (100)
CD4+TBET+ > EBER– CD20+	0
CD4+TBET+ ≈ EBER– CD20+	0
**TH2 (CD4+CMAF+) CELLS: EBER– CD20+ CELLS**^b^	
CD4+CMAF+ < EBER– CD20+	0
CD4+CMAF+ > EBER– CD20+	11 (73.3)
CD4+CMAF+ ≈ EBER– CD20+	4 (26.7)
**REGULATORY T (FOXP3+) CELLS: EBER– CD20+ CELLS**^b^	
FOXP3+ < EBER– CD20+	14 (93.3)
FOXP3+ > EBER– CD20+	1 (6.7)
FOXP3+ ≈ EBER– CD20+	0
**CD8+ CELLS: EBER– CD20+ CELLS**^b^	
CD8 < EBER– CD20+	0
CD8 > EBER– CD20+	13 (86.7)
CD8 ≈ EBER– CD20+	2 (13.3)
**CYTOTOXIC (TIA1)+ CELLS: EBER– CD20+ CELLS**^b^	
TIA1+ < EBER– CD20+	8 (53.3)
TIA1+ > EBER– CD20+	7 (46.7)
TIA1+ ≈ EBER– CD20+	0
**CYTOTOXIC (GRANZYME B+ CELLS: EBER– CD20+ CELLS**^b^**)**	
Granzyme B+ < EBER– CD20+	3 (20)
Granzyme B+ > EBER– CD20+	10 (66.7)
Granzyme B+ ≈ EBER– CD20+	2 (13.3)
**DIFFERENTIATED CD8+ (TBET+) CELLS: EBER– CD20+ CELLS**^b^	
CD8+TBET+ < EBER– CD20+	8 (53.3)
CD8+TBET+ > EBER– CD20+	3 (18.8)
CD8+TBET+ ≈ EBER– CD20+	4 (2 6.7)
**NON-DIFFERENTIATED CD8+ (TBET–) CELLS: EBER– CD20+ CELLS**^b^	
CD8+TBET– < EBER– CD20+	2 (13.3)
CD8+TBET– > EBER– CD20+	9 (60)
CD8+TBET– ≈ EBER– CD20+	4 (26.7)
**NON-DIFFERENTIATED CD8+TBET- CELLS: DIFFERENTIATED**	
+ **CD8+TBET CELLS**	
CD8+TBET– < CD8+TBET+	1 (6.3)
CD8+TBET– > CD8+TBET+	13 (81.3)
CD8+TBET– ≈ CD8+TBET+	2 (12.5)
**CD68+pSTAT1+ CELLS: CD68+CMAF+ CELLS**	
M1-like > M2-like	16 (100)
M2-like > M1-like	0
M1-like ≈ M2-like	0
**CD163+pSTAT1+ CELLS: CD163+CMAF+ CELLS**	
M1-like > M2-like	12 (75)
M2-like > M1-like	1 (6.3)
M1-like ≈ M2-like	3 (18.8)

a *For comparison of two cell populations, a predominance of one cell population over the other was considered when cell numbers were at least 1.5x higher*.

b *EBV-negative B cells were evaluated as EBER– CD20+ cells and one case was not available for this analysis (Figure S1)*.

It has been reported that among CD8+ lymphocytes, TBET is a master protein that has a role in the differentiation process of these T cells (37). In order to describe differentiated CD8+ T cells in the tissue microenvironment of IM and get insights into their functional status through correlation analysis, we decided to assess quantitatively the CD8+TBET+ cell subset. From the total CD8+ cells, a median of 35% of cells (from 14 to 72%) expressed TBET (Table 2). Remarkably, in two cases TBET was expressed in approximately 50% of CD8+ cells and, in another one, in 72% of CD8+ cells. CD8+TBET+ cells exhibited a homogenous distribution in the interfollicular region. These cells were also observed in the epithelium of the tonsils as highlighted by the triple IHC assays (Figure 2). Interestingly, many of the CD8+TBET+ cells were observed forming clusters in the post-capillary venules, while only few of these cells were observed in the lymphatic vessels, as exemplified in the Figure 2. Additional illustrative pictures are presented as Figure S3.

#### NK Cells and Macrophages

Using CD56 antibody, an antibody suitable for identifying NK cells in single labeling immunohistochemistry (38), CD56+ NK cells were the least prevalent cell subset in the tissue microenvironment (median 8 cells/mm^2^). See Table 2 for detailed range description.

M1-like macrophages were predominant compared to M2-like macrophages, as disclosed by the numbers of CD68+pSTAT1+ cells (median 115 cells/mm^2^) and CD163+pSTAT1+ cells (median 99 cells/mm^2^), in comparison to CD68+CMAF+ cells (median 23 cells/mm^2^) and CD163+CMAF+ cells (median 49 cells/mm^2^). See Table 2 for detailed description. Considering the ratio between macrophage subpopulations, all cases exhibited more CD68+pSTAT1+ than CD68+CMAF+ macrophages (M1-like polarization). Using CD163 as a macrophage marker, 75% (12/16) of cases showed more CD163+pSTAT1+ than CD163+CMAF+ macrophages (M1-like polarization), only 1 case (6.3%) showed more CD163+CMAF+ than CD163+pSTAT1+ macrophages (M2-like polarization), and 18.8% (3/16) of cases showed similar numbers of CD163+pSTAT1+ and CD163+CMAF+ macrophages (Table 3).

### Immune Response and the Magnitude of EBV Infection

As the numbers of EBV+ cells/mm^2^ and viral loads were variable in IM tonsils, regardless of the methodology used to identify the virus, we hypothesized that differences in the immune response could be related to differences in the magnitude of EBV infection. To investigate this, non-parametric correlations were performed among the numbers of EBV+ cells/mm^2^, the numbers of cells expressing viral proteins/mm^2^ and the viral loads, with the numbers of immune cells evaluated in this study.

First, the absolute numbers of EBER+ cells/mm^2^, EBNA2+ cells/mm^2^, LMP1+ cells/mm^2^, EBNA2- LMP1+ cells/mm^2^ (latency IIa), EBNA2+LMP1- cells/mm^2^ (latency IIb), EBNA2+LMP1+ cells/mm^2^ (latency III) and BZLF1+ cells/mm^2^ showed no statistical correlation with the absolute numbers of CD3+, CD8+, differentiated CD8+TBET+, CD8+TBET-, cytotoxic TIA1+, cytotoxic Granzyme B+, CD4+, Th2-like, Th1-like, regulatory T, CD68+pSTAT1+, CD68+CMAF+, CD163+pSTAT1+, CD163+CMAF+, and EBV-negative B cells/mm^2^.

However, a direct correlation was observed between the numbers of BZLF1+ and of M2-like (CD68+CMAF+) cells/mm^2^ (rho = 0.5, *P* = 0.04; Spearman's correlation). A borderline inverse correlation was observed between the numbers of EBER+ and of CD8+ cells/mm^2^ (rho = −0.5, *P* = 0.054; Spearman's correlation) and between the numbers of EBER+CD20+ and CD56+ NK cells/mm^2^ (rho = −0.44, *P* = 0.09; Spearman's correlation), reinforcing the role of NK cells in the control of primary EBV infection (27).

The absolute numbers of EBNA1+ cells/mm^2^ were inversely correlated with the numbers of EBV-negative B cells/mm^2^ (rho = −0.84, *P* < 0.0005; Spearman's correlation). Considering viral loads, an inverse correlation was observed between the numbers of differentiated CD8+TBET+ cells/mm^2^ and EBV copy numbers/10^6^ cells (rho = −0.71, *P* = 0.002; Spearman's correlation). No other correlations were observed.

As most immune cells showed no correlation with the magnitude of EBV infection in the tissue microenvironment of IM, we next evaluated if tissue microenvironment signatures demonstrate a relationship to parameters indicating the magnitude of EBV infection. For this, we compared the numbers of EBV+ cells/mm^2^ and viral load with the immune cell ratios, arbitrarily defining an excess of 1.5 to indicate predominance of a given cell population over another one (28–30, 35) (Figure S1).

No correlation was observed between the numbers of EBER+, EBER+CD20+, EBNA2+, and BZLF1+ cells/mm^2^, and any of the evaluated ratios. However, higher numbers of EBNA1+ cells were detected in cases with deficit of EBV-negative B cells in relation to cytotoxic T cells, as disclosed by the ratios TIA1+: EBER– CD20+ (median 416 EBNA1+ cells/ mm^2^ for TIA1+ > EBER– CD20+ cells vs. median 310 EBNA1+ cells/mm^2^ for TIA1+ < EBER– CD20+ cells; *P* = 0.028, Man-Whitney test) (Figure 4).

Taking into account viral loads, higher EBV loads were observed in cases with a reduction of Th2-like cells in relation to cytotoxic T cells: median 15,855 copies for CD4+CMAF+ > TIA1+ vs. median 56,364 copies for CD4+CMAF+ < TIA1+ cells (*p* = 0.032, Mann-Whitney test). Similar results were observed when Th2-like cells were compared with CD8+TBET- cells. A median 15,855 copies was observed for CD4+CMAF+ > CD8+TBET- vs. median 59,266 copies for ratio CD4+CMAF+ < CD8+TBET- (*P* = 0.028, Mann-Whitney test) (Figure 4). The balance between CD8+TBET+ and CD8+TBET- cells did not influence the magnitude of EBV infection and the numbers of EBV+ cells expressing EBNA1, EBNA2, LMP1 or BZLF1.

### PD-L1 Expression by EBV-Infected Cells Correlates With Infection of T-Cells

As described above, the absolute numbers of EBV-infected T cells were not correlated with the magnitude of EBV infection, immune cell composition of the tissue microenvironment or tissue microenvironment signature. We therefore decided to evaluate if EBER+ cells express the immune regulatory PD-L1 protein, previously shown to be up-regulated in EBV-associated tumors and able to modulate the immune response (39–41).

**Figure 3:**
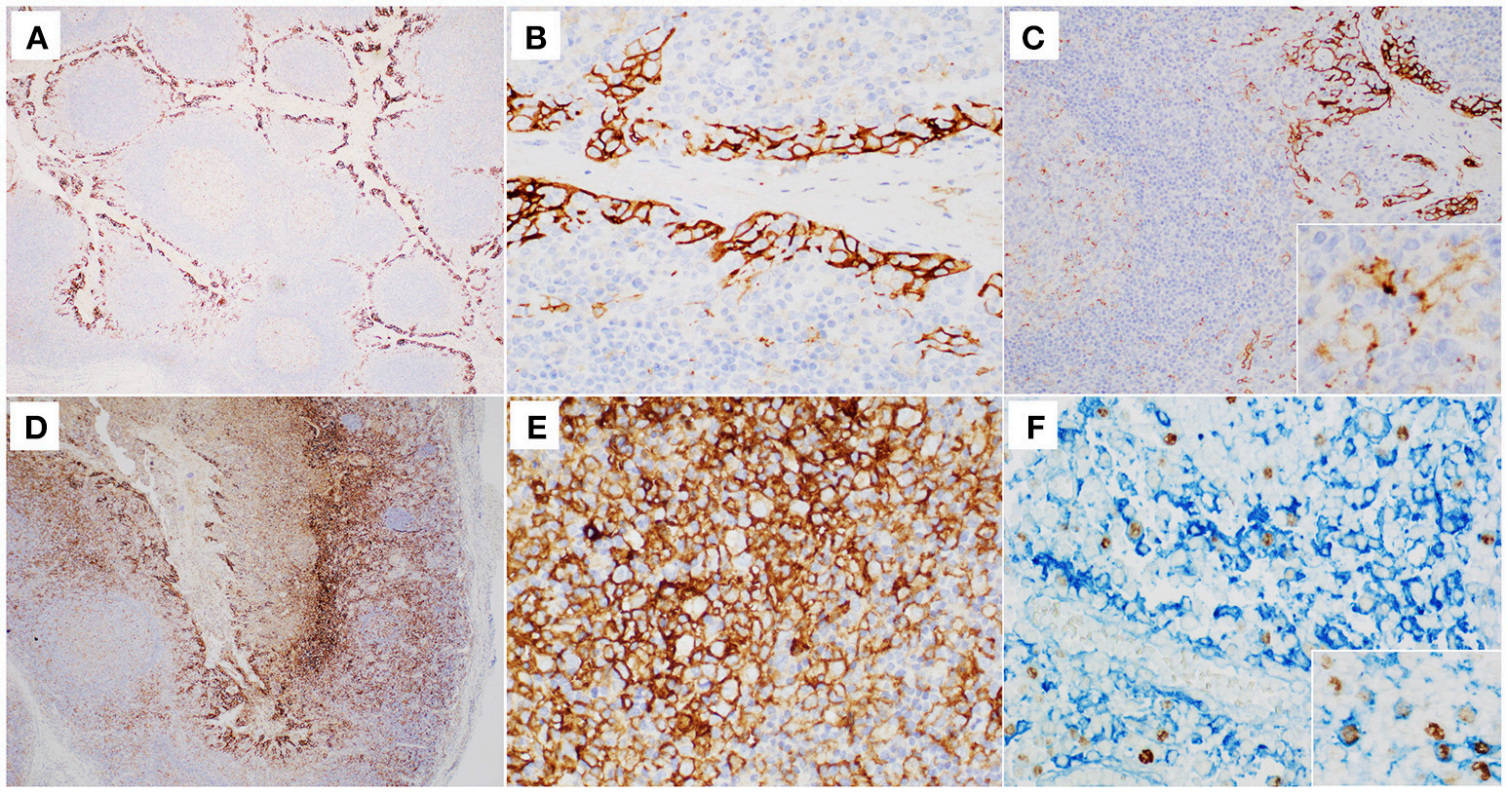
**(A–C)** Single labeling immunohistochemistry (IHC) for the detection of PD-L1 in tonsils with non-specific follicular hyperplasia. The majority of PD-L1+ cells (strong membranous brown staining) were epithelial cells **(A,B)**. In detail **(C)**, only few immune cells, predominantly localized in the germinal center, were PD-L1+ and they exhibited weak membranous brown staining (original magnification **(A)**: 40x; **B**: 400x; **(C)**: 200x and 600x]. **(D,E)** Single labeling IHC in tonsils with diagnosis of IM. Besides epithelial cells, many immune cells were PD-L1+ showing strong membranous brown staining (original magnification 100x and 400x, respectively). **(F)** Double labeling EBER-specific *in situ* hybridization (nuclear brown staining) and PD-L1 IHC (membranous blue staining) reveals that a proportion of PD-L1+ cells is EBV-infected (original magnification 400x and 600x, respectively).

The median of EBER+PD-L1+ cells was 41 cells/mm^2^ (from 11 to 117 cells/mm^2^). Considering all EBER+ cells a median of 14.34% EBER+ cells expressed PD-L1 (from 3.54 to 61.19%) (Figure 3, Table 1). In all cases, the majority of PD-L1+ cells was EBER– (Figure 3F). The numbers of EBER+CD3+ cells/mm^2^ and the numbers of EBER+CD8+ cells/mm^2^ were directly correlated with the numbers of EBER+PD-L1+ cells/mm^2^ (rho = 0.54, *P* = 0.032, and rho = 0.53 *P* = 0.033; Spearman's correlation, respectively).

**Figure 4:**
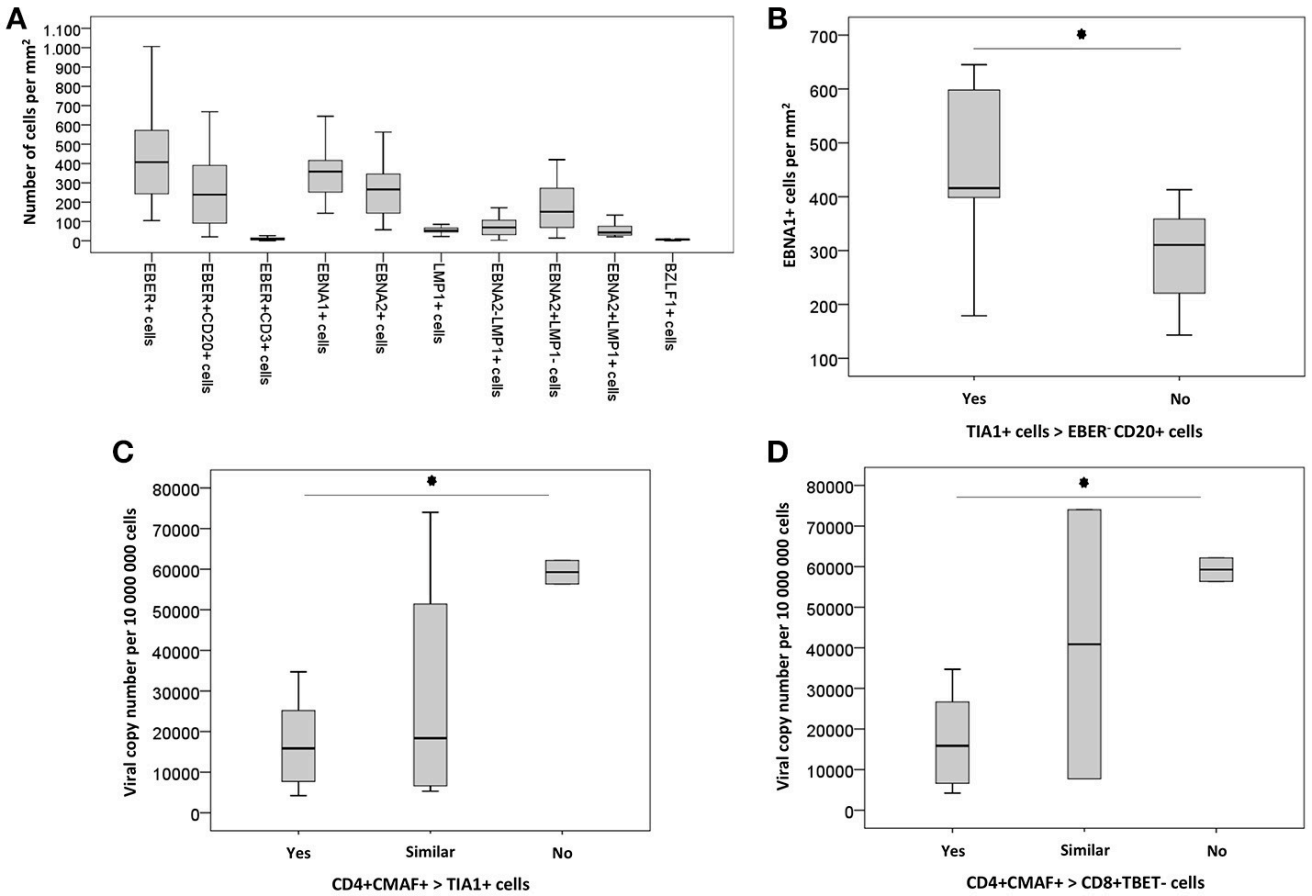
**(A)** Box-plot graphic showing the numeric distribution of EBV infected cells/mm^2^, according to the immunoexpression of viral gene products. **(B)** Box-plot graphic with the numerical distribution of EBNA1+ cells/mm^2^ according to the ratio between cytotoxic TIA1+ and EBER– CD20+ cells. Asterisk indicates *p*-value less than 0.005. **(C)** Box-plot graphic with the numerical distribution of viral copy number per 10^6^ cells according to the ratio between Th2 (CD4+CMAF+) and cytotoxic TIA1+ cells. Asterisk indicates *p*-value lesser than 0.005 between “yes” and “no.” **(D)** Box-plot graphic with the numerical distribution of viral copy number per 10^6^ cells according to the ratio between Th2 (CD4+CMAF+) and not differentiated CD8+TBET- cells. Asterisk symbol indicates *p*-value lesser than 0.005 between “yes” and “no”.

The numbers of EBER+PD-L1+ cells/mm^2^ were statistically correlated neither with the numbers of EBER+, EBER+CD20+, EBNA1+, EBNA2+, and BZLF1+ cells/mm^2^, nor with the viral load. Moreover, when the latency patterns were considered (EBNA2+LMP1+, EBNA2+LMP1-, and EBNA2-LMP1+ cells), no correlations were identified with EBER+PD-L1+ cells. However, a direct correlation was observed between the numbers of EBER+PD-L1+ and LMP1+ cells/mm^2^ (rho = 0.52, *P* = 0.036; Spearman's correlation), consistent with a role for LMP1 in regulating PD-L1 expression (42). Immune cell populations and tissue microenvironment signatures were not correlated with the absolute numbers of EBER+PD-L1+ cells/mm^2^.

## Discussion

Although EBV infection in infectious mononucleosis and anti-viral immune responses have been extensively studied, information regarding the local immune response and characterization of EBV-infected cells in tonsils, i.e., the site of primary EBV infection, are still incomplete (4, 7, 13, 14, 43, 44).

In this work we have addressed these issues through a combined *in situ* and molecular analysis. Considering EBV-infected cells, we show for the first time directly, that approximately 50% of the B cell populations in IM tonsils are infected by EBV. This observation is in line with previous estimates in peripheral blood based on quantitative PCR data (16). We further confirm a previous study showing that EBER+ cells outnumber EBNA2+ and LMP1+ cells. It has been demonstrated previously, that viral latency in acute IM tonsils is not restricted to latency III (14, 18). Here, we extend this observation by showing that EBNA2+/LMP1- latency IIb-type cells are more prevalent than EBNA2-/LMP1+ latency IIa-type cells and EBNA2+/LMP1+ latency III-type cells. These results confirm that heterogeneous viral latency patterns are prevalent in IM tonsils independently of the magnitude of EBV infection. Possibly, these different patterns of viral latency reflect a sequence of events with latency IIb cells representing cells early after EBV infection while latency III and IIa cells may represent later stages of infection (18). Considering that establishment of viral latency is a dynamic process, our approach cannot capture the kinetics of expression and silencing of viral proteins during the disease course leading to EBV persistence. Specifically, it is likely, that latency patterns in individual cells may change during the course of infection within individual cells. Also, it would be difficult to ascertain the exact time point in the natural course of the disease at which tonsillectomy has taken place which would be required to obtain a more dynamic view of the process leading from primary infection to asymptomatic persistence.

Unexpectedly, there was no correlation between the numbers of EBER+ cells and EBNA1+ cells. This may be due to a lesser sensitivity of EBNA1 immunohistochemistry for detecting EBV-positive cells in comparison to EBER-specific *in situ* hybridization, that is comparable to PCR-based methodology (45). Alternatively, it may reflect the establishment of a latency type characterized by expression of the EBERs only. A multi-staining *in situ* approach is necessary to test this hypothesis.

In our IHC approach, we have not used plasma cell differentiation markers to identify EBV-infected plasma cells. The question of plasma cell differentiation of EBV-infected cells in IM was evaluated previously in depth by one of us (GN). In that study it has been shown that variable numbers of EBER+ cells and many BZLF1+ cells exhibited evidence of plasma cell differentiation (14).

Although B cells are the major target of EBV infection, the association of EBV with T cell lymphoproliferative disorders and T/NK cell lymphomas is well described (46–48). In this work, we show that most but not all EBER+ cells expressed the CD20 B-cell antigens. Characterizing the EBER+CD20-negative population further, we observed small numbers of EBER+ cells co-expressing CD3, CD4, or CD8.

Three previous studies reported low proportions (< 10%) of tonsillar EBER+ cells expression T or NK cell markers in IM (13, 49) or tonsils showing non-specific hyperplasia (50). We extend these results by showing that larger proportions of EBER+ cells in IM tonsils showed expression of CD8 as compared to CD4 (6 vs. 3.6%, respectively), in contrast to what has been observed in hyperplastic tonsils, where CD4+ predominated over CD8+ cells (50).

Our results are in line with the observation that the majority of EBV-associated T-lymphoproliferative disorders occurring in childhood displays a CD8+ phenotype (51–53) and raises the possibility that EBV infection of CD8+ T cells during primary infection may indeed be a direct contributory factor to the development of these disorders. In addition to the expression of the EBERs in T-cells, we also demonstrate that subsets of EBV-positive T-cells may express EBNA1 and EBNA2. Co-expression of LMP1 could not be analyzed for technical reasons since reliable detection of two antigens located on the cellular membrane is not feasible with our immunoenzymatic approach. No expression of BZLF1 was observed in T cells suggesting that the vast majority of EBV-infected T cells in IM maintain latent viral infection. It is tempting to propose a possible role of T cells as minor reservoir of EBV. Nonetheless, at present our data are not sufficient to support this claim nor add evidence to the hypothesis that type 2 EBV may establish latency or a prolonged transient infection in the T cell compartment, as suggested by Coleman et al. (54).

EBV utilizes several entry receptors and has evolved multiple mechanisms to enter cells, involving both direct virus–cell contact and cell-to-cell spread of virus (55–59). Although knowledge of B cell and epithelial cell entry receptors and mechanisms has been refined in the last decades, the mechanisms by which EBV infects T cells remain undefined. Previous studies have shown that normal T cells express the receptor CD21/CR2 (60), and about 30–50% of peripheral CD8+ T lymphocytes bind EBV, in contrast to the CD4+ subpopulation (61). However, CD21 on T cells may be different from CD21 on B cells, not allowing an efficient entry (61, 62). Recently, Coleman et al. showed that EBV type 2 displays a unique tropism for CD8+ T cells, being able to efficiently infect and activate them, inducing a distinct EBV gene expression program (63). Our statistical analyses did not identify any association of EBV type, viral loads, numbers of EBV-infected B cells/mm^2^ in the tissue microenvironment or of a particular local immune response with the infection of T cells by EBV. Notably, we did not observe any infection with EBV type 2 in our series.

It has been also hypothesized that EBV infection may occur at the immunological synapse, during the cytotoxic attack of EBV-infected cells by NK cells or T cells (64), and a local immune interaction between EBV-infected B cells and T cells following direct contact may be plausible. Based on this hypothesis, we decided to evaluate the expression of PD-L1 in EBV-infected cells in IM tonsils.

PD-L1 and PD-L2 are ligands of the inhibitory receptor PD-1, and are members of the CD28 and B7 families of proteins involved in the regulation of T cell activation and tolerance (65–68). PD-L1 has been shown to be up-regulated by LMP1 and interferon-γ pathway (42).

In tonsils with non-specific follicular hyperplasia, only scarce lymphocytes weakly expressed PD-L1. By contrast in IM, large numbers of lymphocytes showed strong expression of PD-L1. In parts this is due to the up-regulated expression of PD-L1 in EBV+ B-cells, likely mediated through LMP1 and/or EBNA2 (42, 69). The bulk of PD-L1+ cells, however, was EBV-negative. Thus, the increased expression of PD-L1 in IM vs. non-specific tonsillar hyperplasia may be a consequence of the type of Th1/cytotoxic oriented immune response in IM, where inhibitory circuits may be homeostatically activated.

The expression of PD-L1 by EBV-infected cells was variable and a positive correlation between the numbers of EBER+PD-L1+ and EBER+CD3+ cells/mm^2^ was observed. Elucidating the causes underlying this association is beyond the methodological scope of this study. However, it is possible to hypothesize that immunological synapses involving the ligation of PD-L1 with PD-1 in T cells in IM tonsils may directly or indirectly contribute to the infection of T cells by EBV, either favoring a window for cell-to-cell infection, or generating conditions for EBV-infected T cell to evade immunological control. PD-1 expression exhibits a particular profile in acute IM, being upregulated on EBV-specific CD8+ T cells and directly correlated with viral load in the periphery (70). PD-1 expression was also shown to be epitope- and T-cell receptor usage specific (70). Further functional studies are warranted to understand the mechanisms of EBV T cell infection.

An extensive characterization of the immune cells in the tissue microenvironment of IM has not yet been published. Unlike the situation in peripheral blood (71), we did not observe a predominance of CD8+ over CD4+ cells in the tissue microenvironment of IM. This result is in line with the previously reported poor homing of CD8+ cells to the tonsils in the acute phase of IM (15). In addition, an enhanced migration of EBV-specific CD8+ T cells from tonsils to peripheral blood, after recognition of cognate antigen, may account for this observation and may explain the large numbers of CD8+TBET+ cells in the tissue blood vessels observed in the present work. An investigation of integrin and selection profiles expressed by interfollicular vs. intravessel EBV-specific CD8+ T cells in IM tonsils could help to shed light onto this question. Alternatively or additionally, a local expansion of CD4+ cells cannot be rule out.

In tonsils, the initial contact between CD8+ T cells and EBV-infected B-cells may result in the differentiation of CD8+ T cells and the development of an effective immune response against EBV (10, 15). Based on this premise, we decided to evaluate differentiated CD8+ T cells expressing TBET in the tissue microenvironment of IM (37, 72). In the present work focused on the acute phase of IM, a variable proportion of CD8+TBET+ cells ranging from 14 to 72% of total CD8+ T-cells and an inverse correlation between the numbers of these cells and viral load were observed. These features suggest a contribution of CD8+TBET+ cells to the control of primary EBV infection and support a role of TBET as activation marker in the CD8+ T cells in tonsils during primary EBV infection.

Interestingly many of the CD8+TBET+ cells were found in the post-capillary venules (leaving the tonsil) and only sparsely in the lymphatic vessels, suggesting that priming and differentiation of a group of CD8+ T cells takes place in the tonsils with subsequent migration into peripheral blood. Additionally many of CD8+ T cells observed in the lymphatic vessels did not express TBET. Considering these features, the numeric variability of CD8+TBET+ cells in the IM tissue microenvironment and the inverse correlation of the numbers of these cells with viral load, it is tempting to speculate that this subset of CD8+ T cells may play a pivotal role in anti-EBV immune response in IM.

Many studies have been described the hierarchical antigenicity of EBV proteins in the IM (4, 5, 20). The numbers of EBNA1-expressing cells showed a strong inverse correlation with the amount of EBV– B cells (rho = −0.84). While this might reflect recovery from disease with decline of the numbers of EBV+ B cells accompanied by an increase of the fraction of EBV– B cells, the lack of correlations of absolute numbers of EBV– B cells with viral load and numbers of EBER+, EBER+CD20+, EBNA2+, and LMP1+ cells/mm^2^ do not support this hypothesis. We have also considered the possibility that the relative increase in the number of EBNA1- B-cells in some cases may reflect the emergence of presumably EBV-negative B-cells producing EBNA1-specific antibodies in the course of IM. This appears unlikely, however, since EBNA1-specific antibodies are not detected in acute IM, and take several months to appear (71). This slow kinetics has been linked to delayed CD4+ T cell responses to EBNA1 as consequence of limited access of this protein for class II MHC processing (71). In IM, the role of B cells as antigen presenting cell (APC) of endogenous viral antigens (to be recognized by specific CD4^+^T cells) has not been addressed so far. Three observations from our study suggest a role for B cells and Th2 cells in controlling EBV infection: (i) the direct correlation of numbers of EBV-negative B cells and Th2-like cells (rho = 0.67); (ii) the direct correlation between the numbers of EBNA1-expressing cells with a low ratio of EBV– B cells over cytotoxic T cells; and (iii) the association of a low ratio of Th2-like cells over cytotoxic T cells with a high viral load. Further studies are required to check our hypothesis and to evaluate the role of B cells in the immune control of primary EBV infection in the tonsils, as well as the pathways involved for this function. Given their recently defined importance in the immune processes underlying IM (73) and their potential as therapeutic tools (74, 75), will be relevant to evaluate CD4+ T cells with cytotoxic activity (76) in IM and their distribution in the tissue microenvironment, considering the localization of EBV-infected cells.

The molecule CD83, a marker used to identify mature dendritic cells, participates in the regulation of T- and B-cell lymphocyte maturation and in the regulation of their peripheral responses (77, 78). On account of this, we hypothesized that the expression of CD83 by EBV-infected B-cells could influence the microenvironment composition. However, no correlation between the numbers of EBER+CD83+ cells and the immune cell composition was observed. A direct correlation of LMP1+ cells with EBER+CD83+ and PAX5+CD83+ cells, together with the absence of correlations of LMP1+ cells, with EBER– CD83+ cells, highlights the role of LMP1 in the aberrant expression of CD83 by the EBV+ B cells, as described *in vitro* (36).

In summary, we show that EBV is present in approximately 50% of B-cells in IM showing heterogeneous patterns of latent viral gene expression probably reflecting different stages of infection. While the vast majority of EBV+ cells are B-cells, around 9% express T-cell antigens, with a predominance of CD8+ over CD4+ cells. Our results point to a possible role for PD-L1 in EBV infection of T-cells. The microenvironment of IM tonsils was characterized by a predominance of M1-polarized macrophages over M2-polarized cells. However, at the T-cell level, a heterogeneous picture emerged with numerous Th1/cytotoxic cells accompanied and sometimes outnumbered by T2/regulatory T-cells. Further, we provide evidence suggesting a contribution of B- and Th2-like cells to the control of primary EBV infection. Thirty-five percent of CD8+ cells were differentiated CD8+TBET+ cells, frequently detected in post-capillary venules. An inverse correlation was observed between the numbers of CD8+TBET+ cells and viral load suggesting a pivotal role for these cells in the control of primary EBV infection. Our results provide the basis for a better understanding of immune reactions in EBV-associated tumors.

## Author Contributions

MB designed and performed experiments, collected and analyzed data and wrote the manuscript. GV-L performed experiments and analyzed data. PS performed experiments. RH designed experiments, analyzed data and co-wrote the manuscript. GN designed experiments and co-wrote the manuscript.

### Conflict of Interest Statement

The authors declare that the research was conducted in the absence of any commercial or financial relationships that could be construed as a potential conflict of interest.
