# Supplementary material for: Revisiting the Tissue Microenvironment of Infectious Mononucleosis: Identification of EBV Infection in T Cells and Deep Characterization of Immune Profiles
Source: Front Immunol. 2019 Feb 20;10:146. doi: 10.3389/fimmu.2019.00146 (PMC6391352; doi:10.3389/fimmu.2019.00146)
Supplement: Supplementary file 1 [file Table_1.DOC]

Supplementary Material

**Revisiting the Tissue Microenvironment of Infectious Mononucleosis: Identification of EBV Infection in T Cells and Deep Characterization of Immune Profiles**

Mário Henrique M. Barros*^1^, Gabriela Vera-Lozada^2^, Priscilla Segges^2^, Rocio Hassan^2^, Gerald Niedobitek^1,3^

^1^Institute for Pathology, Unfallkrankenhaus Berlin, Berlin, Germany.

^2^Bone Marrow Transplantation Center, Instituto Nacional de Câncer (INCA), Rio de Janeiro, Brazil.

^3^Institute for Pathology, Sana Klinikum Lichtenberg, Berlin, Germany.

**Address for Correspondence:**

Mário Barros, Institute for Pathology, Unfallkrankenhaus Berlin, Warener Str. 7, 12683 Berlin, Germany. Phone: +49 30 5681 3750. Fax: +49 30 5681 3753. E-mail: [mariohenrique.barros@gmail.com](mailto:mariohenrique.barros@gmail.com)

Table S1. Antibodies used for immunohistochemical staining.

| **Antibody** | **Clone** | **Source** | **Buffer Retrieval** |
| --- | --- | --- | --- |
| CD20 | L26 | DakoCytomation | Citrate |
| PAX5 | DAK-Pax5 | DakoCytomation | EDTA |
| CD3 | Polyclonal | DakoCytomation | EDTA |
| CD4 | 1F6 | Novocastra | EDTA |
| FOXP3 | 22510 | Abcam | EDTA |
| CMAF | M-153 | Santa Cruz | EDTA |
| TBET | 4B10 | Santa Cruz | EDTA |
| CD8 | C8/144B | DakoCytomation | EDTA |
| TIA1 | TIA-1 | Abcam | EDTA |
| Granzyme B | GrB-7 | DakoCytomation | EDTA |
| CD68 | PG-M1 | DakoCytomation | EDTA |
| CD163 | 10D6 | Novocastra | EDTA |
| CD83 | 1H4b | Novocastra | EDTA |
| PD-L1 | QR1 | Quartett | EDTA |
| LMP1 | CS 1-4 | DakoCytomation | EDTA |
| BZLF1 | BZ1 | Santa Cruz | EDTA |
| EBNA1 | 1H4 | gift from Dr. Kremmer | EDTA |
| EBNA2 | PE2 | gift from Dr. Rowe | EDTA |
| Factor VIII | EP3372 | Zytomed Systems | EDTA |
| CK5,6 | D5/16B4 | Zytomed Systems | EDTA |
| Podoplanin | D2-40 | BioLegend | EDTA |

**Table S2**: Correlations among the cell populations evaluated in this study.

|  | **EBER+** | **EBNA1+** | **EBNA2+** | **LMP1+** | **BZLF1+** | **Lat. IIa** | **Lat. IIb** | **Lat. III** | **EBER+CD20+** | **EBER+CD3+** | **EBER+CD4+** | **EBER+CD8+** | **EBER+CD83+** | **EBER+PD-L1+** | **CD3+** | **CD4+** | **CD4+CMAF+** | **FOXP3+** | **CD4+TBET+** | **CD8+** | **CD8+TBET+** | **CD8+TBET-** | **TIA1+** | **GRANZYME B+** | **EBER- CD20+** | **EBER- CD83+** | **PAX5+CD83+** | **CD56+** | **CD68+PSTAT1+** | **CD68+CMAF+** | **CD163+PSTAT1+** | **CD163+CMAF+** |
| --- | --- | --- | --- | --- | --- | --- | --- | --- | --- | --- | --- | --- | --- | --- | --- | --- | --- | --- | --- | --- | --- | --- | --- | --- | --- | --- | --- | --- | --- | --- | --- | --- |
| **EBER+** |  | ↑  0.45 | ↑  0.007 | ↓  0.55 | ↑  0.67 | ↓  0.46 | ↑  0.039 | ↑  0.25 | ↑  <0.0005 | ↑  0.83 | ↑  0.53 | ↓  0.31 | ↑  0.23 | ↓  0.88 | ↓  0.84 | ↓  0.08 | ↓  0.22 | ↓  0.73 | ↑  0.97 | ↓  0.054 | ↓  0.2 | ↓  0.11 | ↓  0.75 | ↑  0.33 | ↓  0.12 | ↓  0.31 | ↓  0.64 | ↓  0.3 | ↓  0.16 | ↑  0.93 | ↓  0.93 | ↑  0.63 |
| **EBNA1+** | ↑  0.45 |  | ↑  0.03 | ↑  0.66 | ↓  0.9 | ↓  0.007 | ↑  0.031 | ↑  0.13 | ↓  0.84 | ↓  0.2 | ↓  0.3 | ↓  0.45 | ↓  0.96 | ↓  0.93 | ↓  0.88 | ↑  0.33 | ↓  0.36 | ↑  0.56 | ↓  0.84 | ↓  0.31 | ↑  0.78 | ↓  0.18 | ↓  0.51 | ↑  0.59 | ↓  < 0.0005 | ↑  0.66 | ↑  0.87 | ↓  0.44 | ↑  0.45 | ↑  0.37 | ↑  0.015 | ↑  0.002 |
| **EBNA2+** | ↑  0.007 | ↑  0.03 |  | ↑  0.47 | ↑  0.55 | ↓  0.023 | ↑  0.001 | ↑  0.38 | ↑  0.13 | ↓  0.43 | ↓  0.15 | ↑  0.96 | ↑  0.38 | ↑  0.89 | ↑  0.52 | ↓  0.25 | ↓  0.26 | ↓  0.83 | ↑  0.48 | ↓  0.3 | ↓  0.82 | ↓  0.17 | ↓  0.98 | ↓  0.88 | ↓  0.05 | ↓  0.83 | ↑  0.89 | ↑  0.89 | ↓  0.54 | ↓  0.95 | ↑  0.89 | ↑  0.22 |
| **LMP1+** | ↓  0.55 | ↑  0.66 | ↑  0.47 |  | ↑  0.16 | ↑  0.16 | ↑  0.44 | ↑  0.38 | ↓  0.6 | ↑  0.29 | ↓  0.72 | ↑  0.059 | ↑  0.021 | ↑  0.036 | ↓  0.65 | ↓  0.27 | ↓  0.76 | ↑  0.076 | ↑  0.54 | ↑  0.74 | ↓  0.97 | ↑  0.69 | ↓  0.20 | ↑  0.74 | ↓  0.74 | ↓  0.64 | ↑  0.019 | ↑  0.44 | ↓  0.27 | ↑  0.45 | ↓  0.17 | ↓  0.4 |
| **BZLF1+** | ↑  0.67 | ↓  0.91 | ↑  0.55 | ↑  0.16 |  | ↑  0.87 | ↓  0.96 | ↓  0.51 | ↑  0.94 | ↑  0.12 | ↓  0.4 | ↓  0.86 | ↑  0.35 | ↑  0.37 | ↓  0.42 | ↓  0.08 | ↓  0.96 | ↓  0.79 | ↑  0.62 | ↑  0.28 | ↓  0.71 | ↑  0.26 | ↑  0.83 | ↑  0.94 | ↓  0.80 | ↓  0.58 | ↑  0.37 | ↑  0.31 | ↓  0.68 | ↑  0.044 | ↓  0.33 | ↓  0.97 |
| **Lat. IIa** | ↓  0.46 | ↓  0.007 | ↓  0.023 | ↑  0.44 | ↑  0.87 |  | ↓  0.019 | ↓  0.41 | ↑  0.81 | ↑  0.11 | ↑  0.35 | ↑  0.18 | ↑  0.3 | ↑  0.55 | ↓  0.89 | ↓  0.91 | ↑  0.12 | ↑  0.27 | ↑  0.56 | ↑  0.51 | ↓  0.45 | ↑  0.052 | ↓  0.27 | ↓  0.98 | ↑  0.014 | ↑  0.55 | ↑  0.09 | ↓  0.88 | ↓  0.37 | ↓  0.48 | ↓  0.01 | ↓  <0.0005 |
| **Lat. IIb** | ↑  0.039 | ↑  0.031 | ↑  0.001 | ↑  0.91 | ↓  0.96 | ↓  0.019 |  | ↑  0.93 | ↑  0.37 | ↓  0.23 | ↓  0.27 | ↓  0.29 | ↓  0.76 | ↓  0.48 | ↓  0.78 | ↓  0.49 | ↑  0.86 | ↑  0.80 | ↑  0.80 | ↓  0.11 | ↓  0.59 | ↓  0.057 | ↑  0.88 | ↓  0.43 | ↓  0.18 | ↓  0.26 | ↓  0.23 | ↓  0.62 | ↑  0.91 | ↓  0.97 | ↑  0.70 | ↑  0.1 |
| **Lat. III** | ↑  0.25 | ↑  0.13 | ↑  0.38 | ↑  0.38 | ↓  0.51 | ↓  0.41 | ↑  0.93 |  | ↑  0.14 | ↓  0.46 | ↑  0.26 | ↑  0.49 | ↑  0.53 | ↑  0.26 | ↓  0.34 | ↓  0.73 | ↓  0.006 | ↑  0.82 | ↓  0.25 | ↓  0.11 | ↓  0.55 | ↓  0.22 | ↓  0.26 | ↑  0.043 | ↓  0.008 | ↓  0.85 | ↑  0.56 | ↓  0.13 | ↓  0.29 | ↑  0.37 | ↑  0.08 | ↑  0.49 |
| **EBER+CD20+** | ↑  < 0.0005 | ↓  0.84 | ↑  0.13 | ↓  0.60 | ↑  0.94 | ↑  0.81 | ↑  0.37 | ↑  0.14 |  | ↑  0.73 | ↑  0.39 | ↓  0.31 | ↑  0.12 | ↓  0.94 | ↓  0.77 | ↓  0.11 | ↓  0.27 | ↑  0.79 | ↓  0.9 | ↓  0.11 | ↓  0.058 | ↓  0.51 | ↓  0.62 | ↑  0.35 | ↓  0.59 | ↓  0.72 | ↑  0.49 | ↓  0.094 | ↓  0.29 | ↓  0.62 | ↓  0.46 | ↓  0.69 |
| **EBER+CD3+** | ↑  0.83 | ↓  0.20 | ↓  0.43 | ↑  0.29 | ↑  0.12 | ↑  0.11 | ↓  0.23 | ↓  0.46 | ↑  0.73 |  | ↑  0.54 | ↑  0.59 | ↑  0.034 | ↑  0.032 | ↓  0.75 | ↓  0.2 | ↑  0.57 | ↑  0.44 | ↓  0.97 | ↑  0.89 | ↑  0.74 | ↓  0.71 | ↓  0.12 | ↑  0.8 | ↑  0.4 | ↓  0.94 | ↑  0.22 | ↓  0.51 | ↓  0.29 | ↑  0.22 | ↓  0.052 | ↓  0.31 |
| **EBER+CD4+** | ↑  0.53 | ↓  0.3 | ↓  0.15 | ↓  0.72 | ↓  0.4 | ↑  0.35 | ↓  0.27 | ↑  0.26 | ↑  0.39 | ↑  0.54 |  | ↑  0.78 | ↑  0.14 | ↓  0.94 | ↓  0.39 | ↓  0.34 | ↓  0.93 | ↑  0.16 | ↓  0.66 | ↓  0.65 | ↑  0.75 | ↓  0.42 | ↑  0.95 | ↑  0.2 | ↑  0.79 | ↑  0.85 | ↑  0.85 | ↓  0.27 | ↑  0.85 | ↑  0.5 | ↑  0.98 | ↓  0.45 |
| Lat. IIa: latency IIa (EBNA2- LMP1+ cells). Lat. IIb: latency IIb (EBNA2+LMP1- cells). Lat. III: latency III (EBNA2+LMP1+ cells). ↑: direct correlation. ↓: inverse correlation. The numbers represent the P-value from Spearman´s correlation. | | | | | | | | | | | | | | | | | | | | | | | | | | | | | | | | |
| **Table S2 (continuation)**: Correlations among the cell populations evaluated in this study. | | | | | | | | | | | | | | | | | | | | | | | | | | | | | | | | |
|  | **EBER+** | **EBNA1+** | **EBNA2+** | **LMP1+** | **BZLF1+** | **Lat. IIa** | **Lat. IIb** | **Lat. III** | **EBER+CD20+** | **EBER+CD3+** | **EBER+CD4+** | **EBER+CD8+** | **EBER+CD83+** | **EBER+PD-L1+** | **CD3+** | **CD4+** | **CD4+CMAF+** | **FOXP3+** | **CD4+TBET+** | **CD8+** | **CD8+TBET+** | **CD8+TBET-** | **TIA1+** | **GRANZYME B+** | **EBER- CD20+** | **EBER- CD83+** | **PAX5+CD83+** | **CD56+** | **CD68+PSTAT1+** | **CD68+CMAF+** | **CD163+PSTAT1+** | **CD163+CMAF+** |
| **EBER+CD8+** | ↓  0.31 | ↓  0.45 | ↑  0.96 | ↑  0.059 | ↓  0.86 | ↑  0.18 | ↓  0.29 | ↑  0.49 | ↓  0.31 | ↑  0.59 | ↑  0.78 |  | ↑  0.31 | ↑  0.033 | ↓  0.9 | ↓  0.71 | ↓  0.82 | ↑  0.72 | ↑  0.86 | ↓  0.98 | ↑  0.41 | ↓  0.8 | ↓  0.39 | ↑  0.96 | ↑  0.66 | ↑  0.44 | ↑  0.26 | ↑  0.76 | ↓  0.41 | ↑  0.9 | ↓  0.37 | ↓  0.11 |
| **EBER+CD83+** | ↑  0.23 | ↓  0.96 | ↑  0.38 | ↑  0.021 | ↑  0.35 | ↑  0.3 | ↓  0.76 | ↑  0.53 | ↑  0.12 | ↑  0.034 | ↑  0.11 | ↑  0.31 |  | ↑  0.083 | ↑  0.97 | ↓  0.19 | ↓  0.51 | ↑  0.05 | ↑  0.71 | ↓  0.81 | ↓  0.85 | ↓  0.82 | ↓  0.4 | ↑  0.17 | ↓  0.8 | ↑  0.42 | ↑  0.008 | ↓  0.68 | ↓  0.49 | ↑  0.77 | ↓  0.14 | ↓  0.43 |
| **EBER+PD-L1+** | ↓  0.88 | ↓  0.93 | ↑  0.89 | ↑  0.036 | ↑  0.37 | ↑  0.55 | ↓  0.48 | ↑  0.26 | ↓  0.94 | ↑  0.032 | ↓  0.94 | ↑  0.033 | ↑  0.083 |  | ↓  0.092 | ↓  0.65 | ↓  0.19 | ↓  0.8 | ↓  0.27 | ↓  0.69 | ↓  0.88 | ↓  0.61 | ↓  0.53 | ↑  0.26 | ↓  0.73 | ↑  0.38 | ↑  0.17 | ↓  0.61 | ↓  0.25 | ↑  0.25 | ↓  0.2 | ↑  0.97 |
| **CD3+** | ↓  0.84 | ↓  0.88 | ↑  0.52 | ↓  0.65 | ↓  0.42 | ↓  0.89 | ↓  0.78 | ↓  0.34 | ↓  0.77 | ↓  0.75 | ↓  0.39 | ↓  0.9 | ↑  0.97 | ↓  0.092 |  | ↑  0.52 | ↑  0.94 | ↓  0.91 | ↑  0.12 | ↑  0.35 | ↑  0.17 | ↑  0.54 | ↑  0.96 | ↑  0.81 | ↑  0.82 | ↓  0.66 | ↓  0.79 | ↑  0.24 | ↓  0.88 | ↓  0.017 | ↑  0.89 | ↓  0.94 |
| **CD4+** | ↓  0.08 | ↑  0.33 | ↓  0.25 | ↓  0.27 | ↓  0.081 | ↓  0.91 | ↓  0.49 | ↓  0.73 | ↓  0.11 | ↓  0.2 | ↓  0.34 | ↓  0.71 | ↓  0.19 | ↓  0.65 | ↑  0.52 |  | ↓  0.79 | ↓  0.63 | ↑  0.5 | ↑  0.43 | ↑  0.36 | ↑  0.3 | ↑  0.39 | ↑  0.81 | ↓  0.78 | ↑  0.11 | ↑  0.71 | ↑  0.60 | ↑  0.042 | ↓  0.2 | ↑  0.4 | ↑  0.13 |
| **CD4+CMAF+** | ↓  0.22 | ↓  0.36 | ↓  0.26 | ↓  0.76 | ↓  0.96 | ↑  0.12 | ↑  0.86 | ↓  0.006 | ↓  0.27 | ↑  0.57 | ↓  0.93 | ↓  0.62 | ↓  0.51 | ↓  0.19 | ↑  0.94 | ↓  0.79 |  | ↑  0.05 | ↑  0.35 | ↑  0.31 | ↑  0.52 | ↑  0.56 | ↓  0.52 | ↓  0.011 | ↑  0.006 | ↑  0.78 | ↓  078 | ↓  0.4 | ↑  0.47 | ↑  0.92 | ↓  0.24 | ↓  0.16 |
| **FOXP3+** | ↓  0.73 | ↑  0.56 | ↓  0.83 | ↑  0.076 | ↓  0.79 | ↑  0.27 | ↑  0.8 | ↑  0.82 | ↑  0.79 | ↑  0.44 | ↑  0.16 | ↑  0.72 | ↑  0.05 | ↓  0.8 | ↓  0.91 | ↓  0.63 | ↑  0.05 |  | ↑  0.6 | ↓  0.76 | ↓  0.95 | ↓  0.82 | ↓  0.021 | ↓  0.39 | ↑  0.71 | ↑  0.5 | ↑  0.021 | ↓  0.11 | ↑  0.50 | ↑  0.84 | ↓  0.59 | ↓  0.24 |
| **CD4+TBET+** | ↑  0.97 | ↓  0.84 | ↑  0.48 | ↑  0.54 | ↑  0.62 | ↑  0.56 | ↑  0.8 | ↓  0.25 | ↓  0.9 | ↓  0.97 | ↓  0.66 | ↑  0.86 | ↑  0.71 | ↓  0.27 | ↑  0.12 | ↑  0.5 | ↑  0.35 | ↑  0.6 |  | ↑  0.029 | ↑  0.05 | ↑  0.21 | ↑  0.44 | ↓  0.39 | ↑  0.43 | ↓  0.54 | ↑  0.31 | ↑  0.068 | ↑  0.47 | ↓  0.68 | ↓  0.085 | ↑  0.99 |
| **CD8+** | ↓  0.054 | ↓  0.31 | ↓  0.30 | ↑  0.74 | ↑  0.28 | ↑  0.51 | ↓  0.11 | ↓  0.11 | ↓  0.11 | ↑  0.89 | ↓  0.65 | ↓  0.98 | ↓  0.81 | ↓  0.69 | ↑  0.35 | ↑  0.43 | ↑  0.31 | ↓  0.76 | ↑  0.029 |  | ↑  0.013 | ↑  0.001 | ↑  0.028 | ↑  0.98 | ↑  0.1 | ↑  0.69 | ↑  0.78 | ↑  0.12 | ↑  0.43 | ↑  0.54 | ↓  0.31 | ↑  0.76 |
| **CD8+TBET+** | ↓  0.2 | ↑  0.78 | ↓  0.82 | ↓  0.97 | ↓  0.71 | ↓  0.45 | ↓  0.59 | ↓  0.55 | ↓  0.058 | ↑  0.74 | ↑  0.75 | ↑  0.41 | ↓  0.85 | ↓  0.88 | ↑  0.17 | ↑  0.36 | ↑  0.52 | ↓  0.95 | ↑  0.05 | ↑  0.013 |  | ↓  0.88 | ↑  0.32 | ↓  0.98 | ↑  0.87 | ↓  0.99 | ↓  0.62 | ↑  0.55 | ↑  0.25 | ↑  0.31 | ↑  0.84 | ↑  0.16 |
| **CD8+TBET-** | ↓  0.11 | ↓  0.18 | ↓  0.17 | ↑  0.69 | ↑  0.26 | ↑  0.052 | ↓  0.057 | ↓  0.22 | ↓  0.51 | ↓  0.71 | ↓  0.42 | ↓  0.8 | ↓  0.82 | ↓  0.61 | ↑  0.54 | ↑  0.3 | ↑  0.56 | ↓  0.82 | ↑  0.21 | ↑  0.001 | ↓  0.88 |  | ↑  0.15 | ↑  0.76 | ↑  0.084 | ↑  0.4 | ↑  0.29 | ↑  0.13 | ↑  0.9 | ↓  0.5 | ↓  0.25 | ↓  0.37 |
| **TIA1+** | ↓  0.75 | ↓  0.51 | ↓  0.98 | ↓  0.2 | ↑  0.83 | ↓  0.27 | ↑  0.88 | ↓  0.26 | ↓  0.62 | ↓  0.12 | ↑  0.95 | ↓  0.39 | ↓  0.4 | ↓  0.53 | ↑  0.96 | ↑  0.39 | ↓  0.52 | ↓  0.021 | ↑  0.44 | ↑  0.028 | ↑  0.32 | ↑  0.15 |  | ↑  0.68 | ↑  0.47 | ↑  0.72 | ↓  0.21 | ↑  0.076 | ↑  0.21 | ↓  0.81 | ↓  0.84 | ↑  0.16 |
| Lat. IIa: latency IIa (EBNA2- LMP1+ cells). Lat. IIb: latency IIb (EBNA2+LMP1- cells). Lat. III: latency III (EBNA2+LMP1+ cells). ↑: direct correlation. ↓: inverse correlation. The numbers represent the P-value from Spearman´s correlation. | | | | | | | | | | | | | | | | | | | | | | | | | | | | | | | | |
| **Table S2 (continuation)**: Correlations among the cell populations evaluated in this study. | | | | | | | | | | | | | | | | | | | | | | | | | | | | | | | | |
|  | **EBER+** | **EBNA1+** | **EBNA2+** | **LMP1+** | **BZLF1+** | **Lat. IIa** | **Lat. IIb** | **Lat. III** | **EBER+CD20+** | **EBER+CD3+** | **EBER+CD4+** | **EBER+CD8+** | **EBER+CD83+** | **EBER+PD-L1+** | **CD3+** | **CD4+** | **CD4+CMAF+** | **FOXP3+** | **CD4+TBET+** | **CD8+** | **CD8+TBET+** | **CD8+TBET-** | **TIA1+** | **GRANZYME B+** | **EBER- CD20+** | **EBER- CD83+** | **PAX5+CD83+** | **CD56+** | **CD68+PSTAT1+** | **CD68+CMAF+** | **CD163+PSTAT1+** | **CD163+CMAF+** |
| **GRANZYME B+** | ↑  0.33 | ↑  0.59 | ↓  0.88 | ↑  0.74 | ↑  0.94 | ↓  0.98 | ↓  0.43 | ↑  0.043 | ↑  0.35 | ↑  0.8 | ↑  0.2 | ↑  0.96 | ↑  0.17 | ↑  0.26 | ↑  0.81 | ↑  0.81 | ↓  0.011 | ↓  0.39 | ↓  0.39 | ↑  0.98 | ↓  0.98 | ↑  0.76 | ↑  0.68 |  | ↓  0.055 | ↓  0.99 | ↓  0.92 | ↓  0.8 | ↓  0.15 | ↑  0.78 | ↑  0.33 | ↑  0.32 |
| **EBER- CD20+** | ↓  0.12 | ↓  < 0.0005 | ↓  0.052 | ↓  0.74 | ↓  0.8 | ↑  0.014 | ↓  0.18 | ↓  0.008 | ↓  0.59 | ↑  0.4 | ↑  0.79 | ↑  0.66 | ↓  0.80 | ↓  0.73 | ↑  0.82 | ↓  0.78 | ↑  0.006 | ↑  0.71 | ↑  0.43 | ↑  0.1 | ↑  0.87 | ↑  0.084 | ↑  0.47 | ↓  0.05 |  | ↑  0.59 | ↑  0.78 | ↑  0.65 | ↑  0.71 | ↓  0.28 | ↓  0.001 | ↓  0.009 |
| **EBER- CD83+** | ↓  0.31 | ↑  0.66 | ↓  0.83 | ↓  0.64 | ↓  0.58 | ↑  0.55 | ↓  0.26 | ↓  0.85 | ↓  0.72 | ↓  0.94 | ↑  0.85 | ↑  0.44 | ↑  0.42 | ↑  0.38 | ↓  0.66 | ↑  0.11 | ↑  0.78 | ↑  0.5 | ↓  0.54 | ↑  0.69 | ↓  0.99 | ↑  0.4 | ↑  0.72 | ↓  0.99 | ↑  0.59 |  | ↑  0.1 | ↓  0.19 | ↑  0.16 | ↓  0.85 | ↑  0.9 | ↑  0.88 |
| **PAX5+CD83+** | ↓  0.64 | ↑  0.87 | ↑  0.89 | ↑  0.019 | ↑  0.37 | ↑  0.093 | ↓  0.23 | ↑  0.56 | ↑  0.49 | ↑  0.22 | ↑  0.85 | ↑  0.26 | ↑  0.008 | ↑  0.17 | ↓  0.79 | ↑  0.71 | ↓  0.78 | ↑  0.021 | ↑  0.31 | ↑  0.78 | ↓  0.62 | ↑  0.29 | ↓  0.21 | ↓  0.92 | ↑  0.76 | ↑  0.1 |  | ↓  0.9 | ↑  0.51 | ↓  0.97 | ↓  0.13 | ↓  0.36 |
| **CD56+** | ↓  0.30 | ↓  0.44 | ↑  0.89 | ↑  0.44 | ↑  0.31 | ↓  0.88 | ↓  0.62 | ↓  0.13 | ↓  0.09 | ↓  0.51 | ↓  0.27 | ↑  0.8 | ↓  0.68 | ↓  0.61 | ↑  0.24 | ↑  0.6 | ↓  0.4 | ↓  0.11 | ↓  0.068 | ↑  0.12 | ↑  0.55 | ↑  0.13 | ↑  0.076 | ↓  0.8 | ↑  0.65 | ↓  0.19 | ↓  0.9 |  | ↑  0.98 | ↓  0.37 | ↓  0.46 | ↓  0.86 |
| **CD68+PSTAT1+** | ↓  0.16 | ↑  0.45 | ↓  0.54 | ↓  0.27 | ↓  0.68 | ↓  0.37 | ↑  0.91 | ↓  0.29 | ↓  0.29 | ↓  0.29 | ↑  0.85 | ↓  0.41 | ↓  0.49 | ↓  0.25 | ↓  0.88 | ↑  0.042 | ↑  0.47 | ↑  0.5 | ↑  0.47 | ↑  0.43 | ↑  0.25 | ↑  0.9 | ↑  0.21 | ↓  0.15 | ↑  0.71 | ↑  0.16 | ↑  0.51 | ↑  0.98 |  | ↓  0.92 | ↑  0.56 | ↑  0.12 |
| **CD68+CMAF+** | ↑  0.93 | ↑  0.37 | ↓  0.95 | ↑  0.45 | ↑  0.044 | ↓  0.48 | ↓  0.97 | ↑  0.37 | ↓  0.62 | ↑  0.22 | ↑  0.50 | ↑  0.9 | ↑  0.77 | ↑  0.22 | ↓  0.017 | ↓  0.2 | ↑  0.92 | ↑  0.84 | ↓  0.68 | ↑  0.54 | ↑  0.31 | ↓  0.5 | ↓  0.81 | ↑  0.78 | ↓  0.28 | ↓  0.85 | ↓  0.97 | ↓  0.37 | ↓  0.92 |  | ↑  0.48 | ↑  0.3 |
| **CD163+PSTAT1+** | ↓  0.93 | ↑  0.015 | ↑  0.89 | ↓  0.17 | ↓  0.33 | ↓  0.01 | ↑  0.79 | ↑  0.084 | ↓  0.46 | ↓  0.052 | ↑  0.98 | ↓  0.37 | ↓  0.14 | ↓  0.2 | ↑  0.89 | ↑  0.4 | ↓  0.24 | ↓  0.59 | ↓  0.085 | ↓  0.31 | ↑  0.84 | ↓  0.25 | ↓  0.84 | ↑  0.33 | ↓  0.001 | ↑  0.9 | ↓  0.13 | ↓  0.46 | ↑  0.56 | ↑  0.48 |  | ↑  0.056 |
| **CD163+CMAF+** | ↑  0.63 | ↑  0.002 | ↑  0.22 | ↓  0.4 | ↓  0.97 | ↓  <0.0005 | ↑  0.1 | ↑  0.49 | ↓  0.69 | ↓  0.31 | ↓  0.45 | ↓  0.11 | ↓  0.43 | ↑  0.97 | ↓  0.94 | ↑  0.13 | ↓  0.16 | ↓  0.24 | ↑  0.99 | ↑  0.76 | ↑  0.16 | ↓  0.37 | ↑  0.16 | ↑  0.32 | ↓  0.009 | ↑  0.88 | ↓  0.36 | ↓  0.86 | ↑  0.12 | ↑  0.3 | ↑  0.05 |  |

Lat. IIa: latency IIa (EBNA2- LMP1+ cells). Lat. IIb: latency IIb (EBNA2+LMP1- cells). Lat. III: latency III (EBNA2+LMP1+ cells). ↑: direct correlation. ↓: inverse correlation. The numbers represent the P-value from Spearman´s correlation.
