# Supplementary figures and images for: Revisiting the Tissue Microenvironment of Infectious Mononucleosis: Identification of EBV Infection in T Cells and Deep Characterization of Immune Profiles
Source: Front Immunol. 2019 Feb 20;10:146. doi: 10.3389/fimmu.2019.00146 (PMC6391352; doi:10.3389/fimmu.2019.00146)

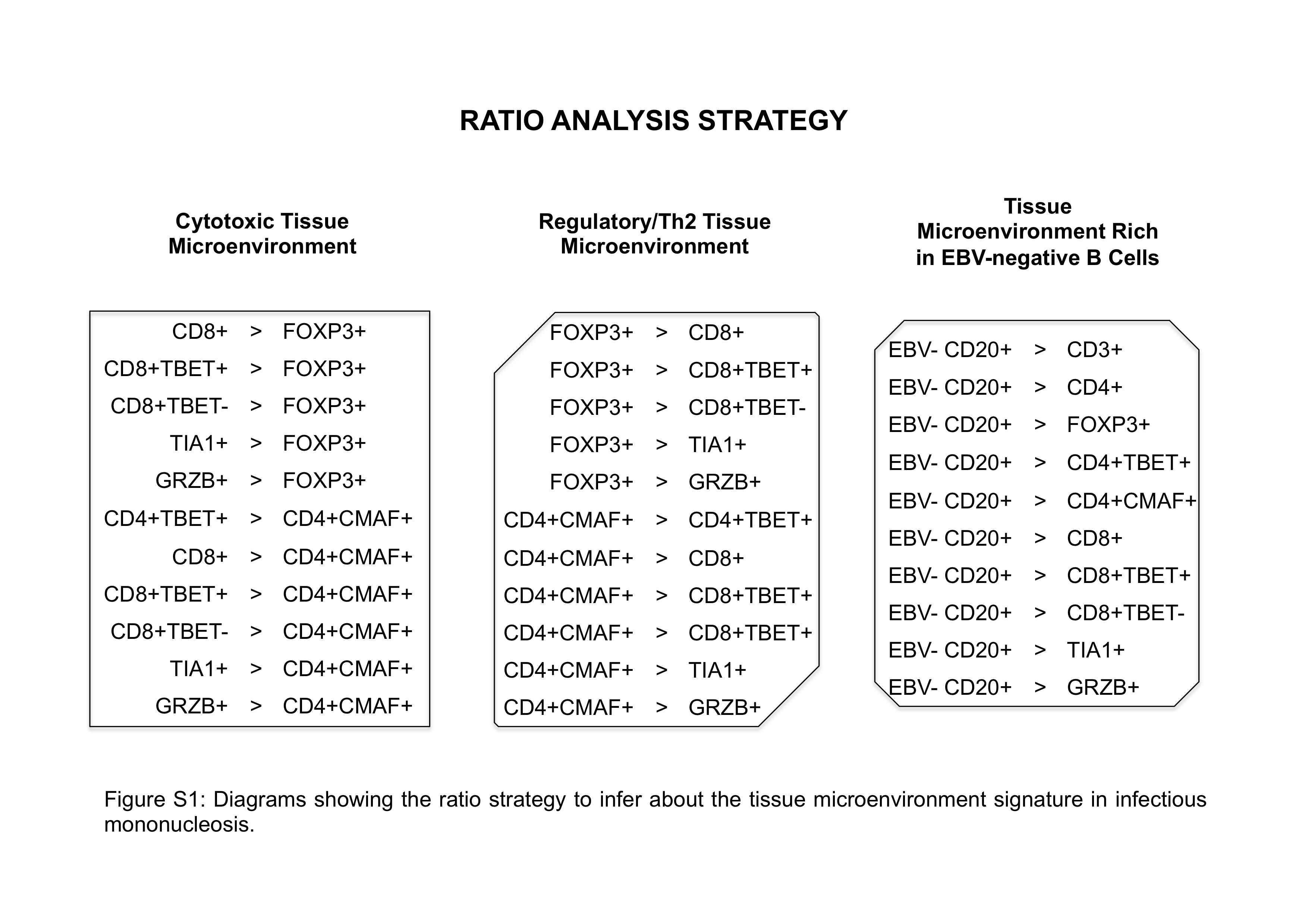

Supplement: Supplementary file 2 [file Image_1.TIFF]

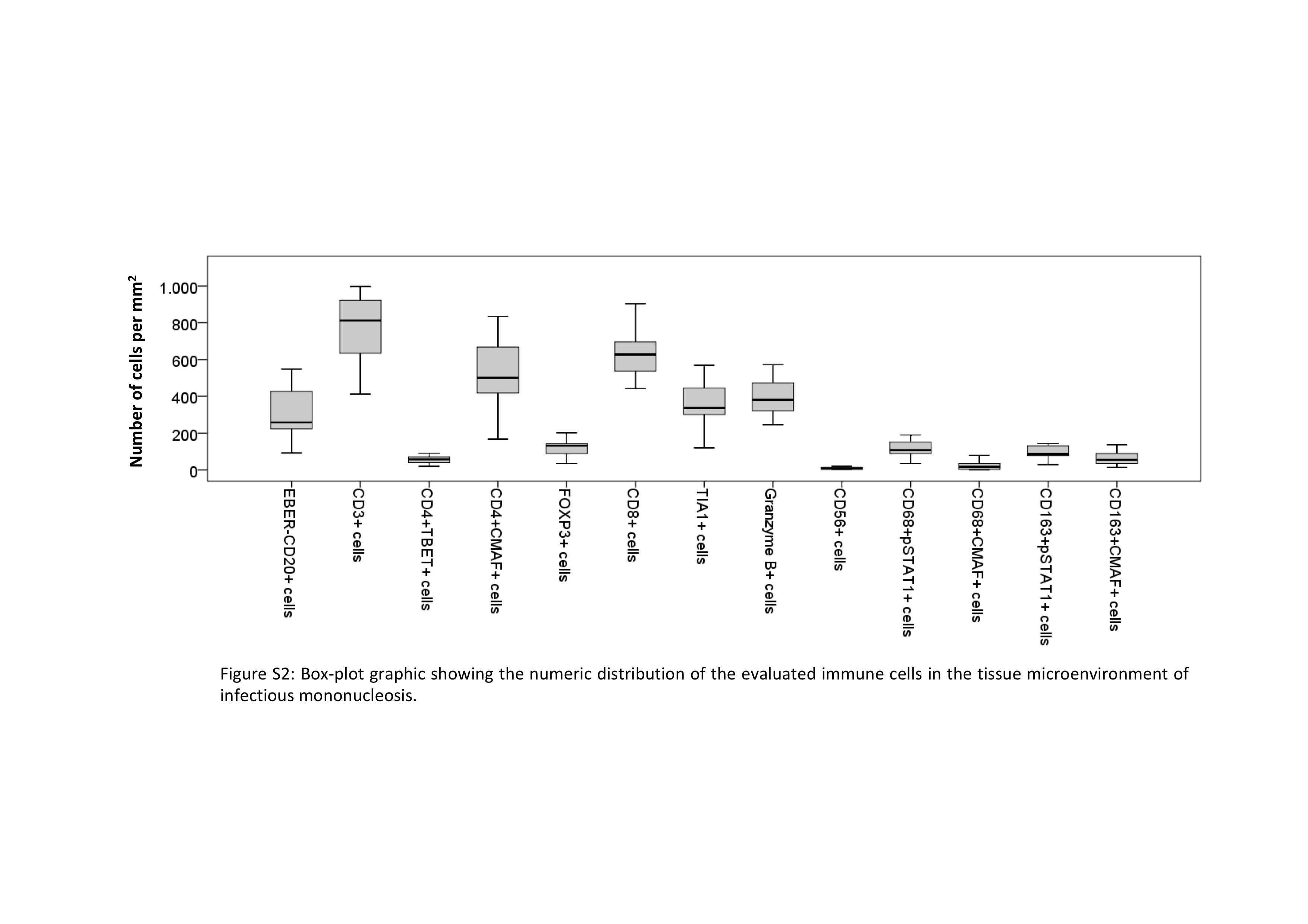

Supplement: Supplementary file 3 [file Image_2.TIFF]
