# Supplementary material for: Revisiting the Tissue Microenvironment of Infectious Mononucleosis: Identification of EBV Infection in T Cells and Deep Characterization of Immune Profiles
Source: Front Immunol. 2019 Feb 20;10:146. doi: 10.3389/fimmu.2019.00146 (PMC6391352; doi:10.3389/fimmu.2019.00146)
Supplement: Supplementary file 4 [file Image_3.pdf]

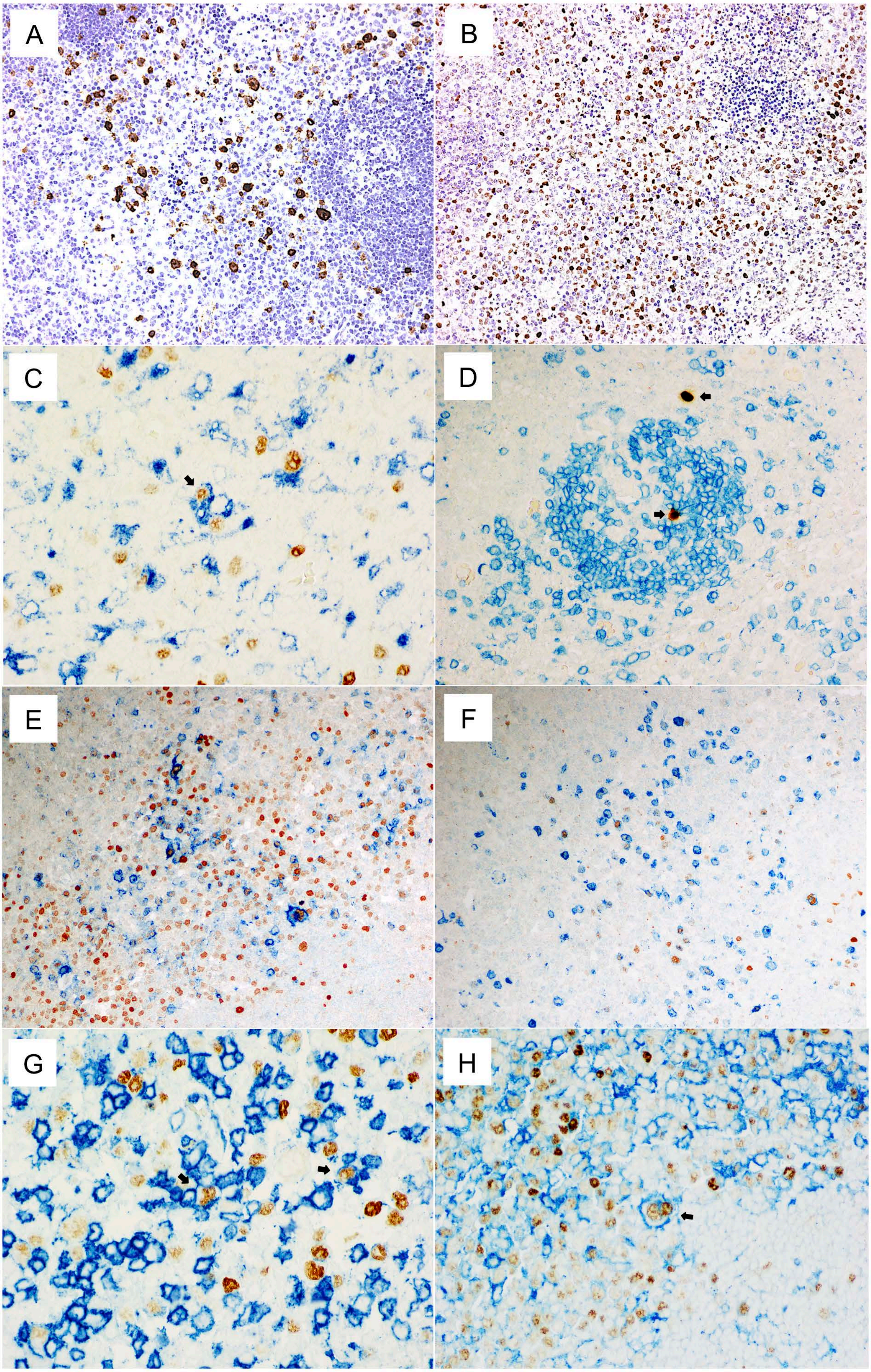
A
B
C
D
E
F
G
H

Legend of Supplemental Figure 3

Two representative cases of IM showing (A) abundant LMP1+ cells (membranous brown staining) and (B) EBNA2+ cells (membranous brown staining) in the interfollicular region (original magnification 400x). (C) Double labeling EBER-specific in situ hybridization (nuclear brown staining) and immunohistochemistry (IHC) for the detection of CD68 (membranous blue staining). Unequivocal EBER+CD68+ cells were not observed (the arrow indicates an EBER+ cell with partial membranous blue staining, original magnification 600x). (D) Double IHC labeling for the detection of BZLF1 (nuclear brown staining) and CD3 (membranous blue staining) showing two BZLF1+CD3- cells (arrows, original magnification 400x). (E, F) Double IHC labeling for the detection of EBNA2 (nuclear brown staining) and LMP1 (membranous blue staining). In (E), EBNA2+LMP1- cells are predominant, while in (F) the predominant cells are EBNA2-LMP1+. In both cases it is possible to identify EBNA2+LMP1+ cells. (G) Double labeling EBER-specific in situ hybridization (nuclear brown staining) and IHC for the detection of CD8 (membranous blue staining). The arrows indicate EBER+CD8+ cells. Note that many EBER+CD8- cells are in contact with EBER-CD8+ cells. (H) Double labeling EBER-specific in situ hybridization (nuclear brown staining) and IHC for the detection of PD-L1 (membranous blue staining). The arrow indicates one EBER+PD-L1+ Reed-Sternberg-like cell.
